# Genome-wide sRNA and mRNA transcriptomic profiling insights into dynamic regulation of taproot thickening in radish (*Raphanus sativus* L.)

**DOI:** 10.1186/s12870-020-02585-z

**Published:** 2020-08-08

**Authors:** Yang Xie, Jiali Ying, Liang Xu, Yan Wang, Junhui Dong, Yinglong Chen, Mingjia Tang, Cui Li, Everlyne M’mbone Muleke, Liwang Liu

**Affiliations:** 1 National Key Laboratory of Crop Genetics and Germplasm Enhancement, Key Laboratory of Horticultural Crop Biology and Genetic Improvement (East China) of MOA, College of Horticulture, Nanjing Agricultural University, Nanjing, 210095 People’s Republic of China; 2 College of Horticulture Science and Technology, Hebei Normal University of Science and Technology, Qinhuangdao, 066004 China; 3 The UWA Institute of Agriculture, and School of Agriculture and Environment, The University of Western Australia, Perth, WA 6009 Australia

**Keywords:** DEGs, DEMs, Radish, RT-qPCR, Taproot thickening

## Abstract

**Background:**

Taproot is the main edible organ and ultimately determines radish yield and quality. However, the precise molecular mechanism underlying taproot thickening awaits further investigation in radish. Here, RNA-seq was performed to identify critical genes involved in radish taproot thickening from three advanced inbred lines with different root size.

**Results:**

A total of 2606 differentially expressed genes (DEGs) were shared between ‘NAU-DY’ (large acicular) and ‘NAU-YB’ (medium obovate), which were significantly enriched in ‘phenylpropanoid biosynthesis’, ‘glucosinolate biosynthesis’, and ‘starch and sucrose metabolism’ pathway. Meanwhile, a total of 16 differentially expressed miRNAs (DEMs) were shared between ‘NAU-DY’ and ‘NAU-YH’ (small circular), whereas 12 miRNAs exhibited specific differential expression in ‘NAU-DY’. Association analysis indicated that miR393a-*bHLH77*, miR167c-*ARF8*, and miR5658-*APL* might be key factors to biological phenomenon of taproot type variation, and a putative regulatory model of taproot thickening and development was proposed. Furthermore, several critical genes including *SUS1*, *EXPB3*, and *CDC5* were characterized and profiled by RT-qPCR analysis.

**Conclusion:**

This integrated study on the transcriptional and post-transcriptional profiles could provide new insights into comprehensive understanding of the molecular regulatory mechanism underlying taproot thickening in root vegetable crops.

## Background

Radish (*Raphanus sativus* L., 2n = 2x = 18) is an important worldwide root vegetable crops belonging to Brassicaceae family. The fleshy taproot is the main product organ and determines the final yield and quality of radish. Abundant nutrient substances exist in fleshy taproot including carbohydrates, crude fiber, vitamin C and protein. Currently, extensive researches on the molecular mechanism of root development had been conducted in a range of plant species such as Arabidopsis [1], tobacco [2], maize [3–5], and rice [6, 7]. However, the molecular mechanism underlying taproot thickening is still far away from being fully clarified in root vegetable crops such as radish.

In the past decades, with the rapid development of ‘omics’ methodology, RNA-seq has become a valuable strategy for systematical identification of differentially regulated genes, miRNAs, and regulation pathways in different tissues, organs, and developmental stages in several plant species, such as *Rosa chinensis* [8], *Glycine max* [9], *Citrus sinensis* [10], *Myrica rubra* [11], *Solanum lycopersicum* [12], and *R. sativus* [13]. Furthermore, the available genome database of radish provided a useful genome information platform for the investigation of molecular mechanism underlying radish taproot thickening [14–16].

In recent years, several studies on identification and dissection of gene expression and complex regulatory network during taproot thickening in radish have been performed. Using RNA-Seq technology, many miRNAs and transcripts were identified to be differentially expressed during radish taproot thickening [13, 17], and carbohydrate metabolism pathway was significantly activated during taproot thickening, particularly in cell proliferating tissues [15]. Nevertheless, previous studies were mainly focused on a single radish cultivar, and the key genes involved in radish taproot thickening among different root-type radish genotypes remains to be accurately identified. Increasing evidences indicated that root shape and size (root-type) are significantly different among different cultivars, and genes related to root-type difference are considered to be candidates that promote or repress taproot thickening in radish [15, 18, 19]. So far, there is no report on identification of genes involved in taproot development in different cultivars, which limits the genetic improvement and germplasm innovation of radish cultivars.

To identify differentially expressed genes (DEGs) and differentially expressed miRNAs (DEMs) involved in taproot thickening, three advanced inbred lines with different root shape and size consisting of radish ‘NAU-DY’ (large acicular), ‘NAU-YB’ (medium obovate) and ‘NAU-YH’ (small circular) were used in this study. An integrated mRNA-seq and sRNA-seq analysis was performed at three development stages of taproot thickening from three advanced inbred lines. Furthermore, RT-qPCR analysis was carried out to validate the expression patterns of several important candidate genes. The outcome of this study could reveal critical genes and miRNAs underlying the taproot thickening, and provide new insights into dynamic regulation of taproot thickening in radish.

## Methods

### Plant materials

**Fig. 1:**
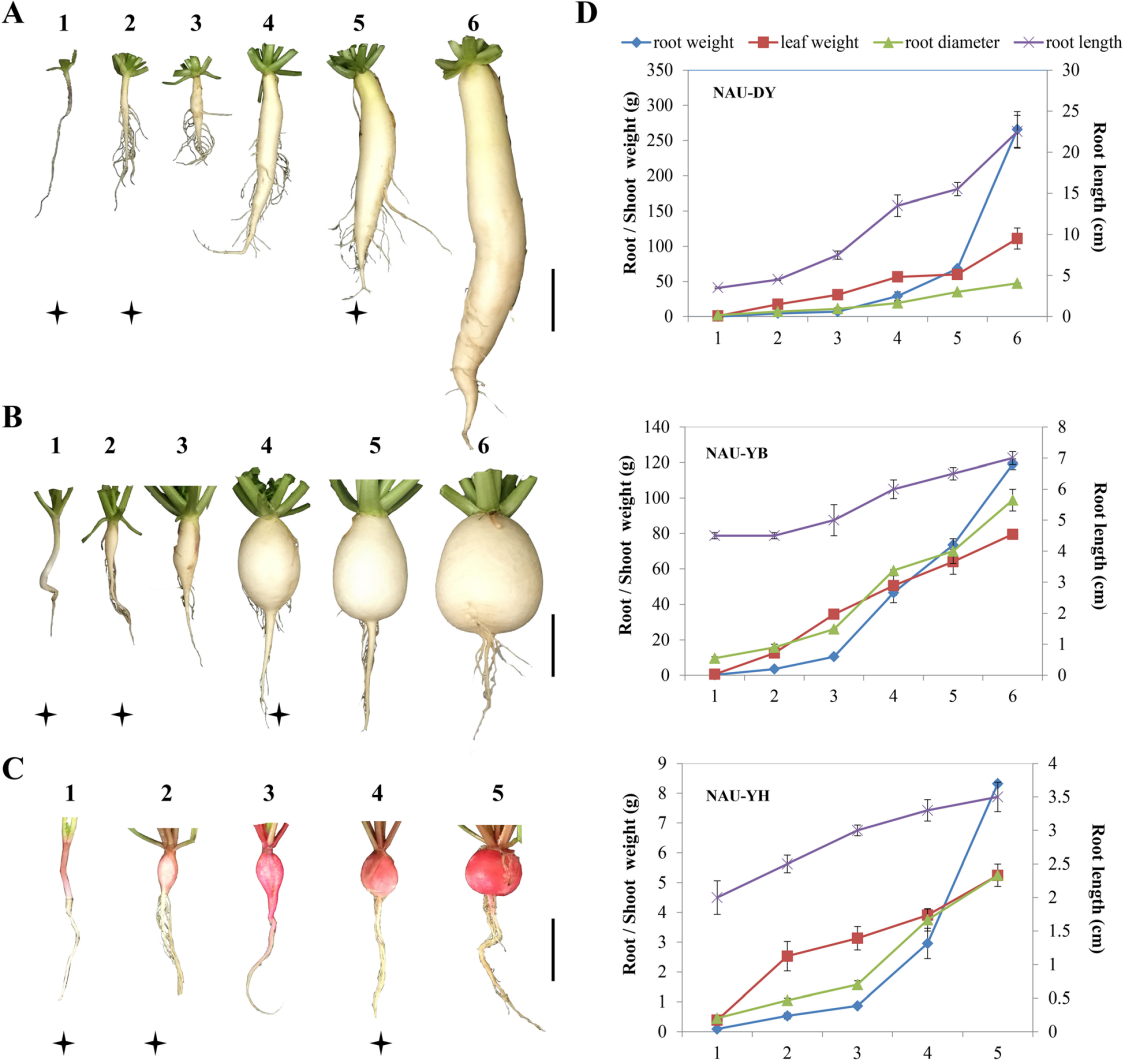
The characteristics of radish growth and development in three advanced lines. **a**. NAU-DY, b. NAU-YB, **c**. NAU-YH, **d**. The growth and development curve of radish. The asterisk represents the plant materials of RNA-Seq, and a bar represents 5 cm. 1 represents pre-cortex splitting stage (PSS), 2 represents cortex splitting stage (CSS) and 3–6 represents expanding stage (ES)

Three advanced inbred lines of radish, ‘NAU-DY’ (large acicular), ‘NAU-YB’ (medium obovate), and ‘NAU-YH’ (small circular), were used in this study, and the seeds were developed from college of Horticulture, Nanjing Agricultural University, Nanjing, China. (Fig. 1). The seeds were germinated on a wet filter paper in darkness at room temperature for 3 days, and then planted in plastic pots and cultured in the growth chamber with 16 h light (25 °C) and 8 h dark (18 °C). For each advanced inbred line, the characteristics of radish growth and development were shown in Fig. 1, and the time point with ‘two leaves and one heart’, cortex splitting, and the highest rate of taproot thickening were as selection criteria of pre-cortex splitting stage (PSS), cortex splitting stage (CSS) and expanding stage (ES), respectively. The taproot samples S1, S2, and S3 were harvested at PSS (‘NAU-DY’, 20 days after sowing, DAS; ‘NAU-YB’, 20 DAS; ‘NAU-YH’, 10 DAS), CSS (‘NAU-DY’, 30 DAS; ‘NAU-YB’, 25 DAS; ‘NAU-YH’, 20 DAS) and ES (‘NAU-DY’, 55 DAS; ‘NAU-YB’, 45 DAS; ‘NAU-YH’, 40 DAS) from five randomly selected individual plants, respectively. Equal amount of samples from five individuals was pooled for library preparation and sequencing. All harvested taproot samples were immediately frozen in liquid nitrogen and stored at − 80 °C for RNA extraction.

### mRNA-seq and sRNA-seq library construction and sequencing

Prior to mRNA library construction, total RNAs were extracted at three different developmental stages of taproot thickening with the TRIzol reagent (Invitrogen) according to the instruction manuals, respectively. The detailed experimental procedures of cDNA library construction and sequencing were performed according to the reported approaches [20, 21]. The corresponding mRNA-seq libraries were named as S1 (‘NAU-DY’, DY_S1; ‘NAU-YB’, YB_S1), S2 (‘NAU-DY’, DY_S2; ‘NAU-YB’, YB_S2), and S3 (‘NAU-DY’, DY_S3; ‘NAU-YB’, YB_S3) library, respectively.

**Fig. 2:**
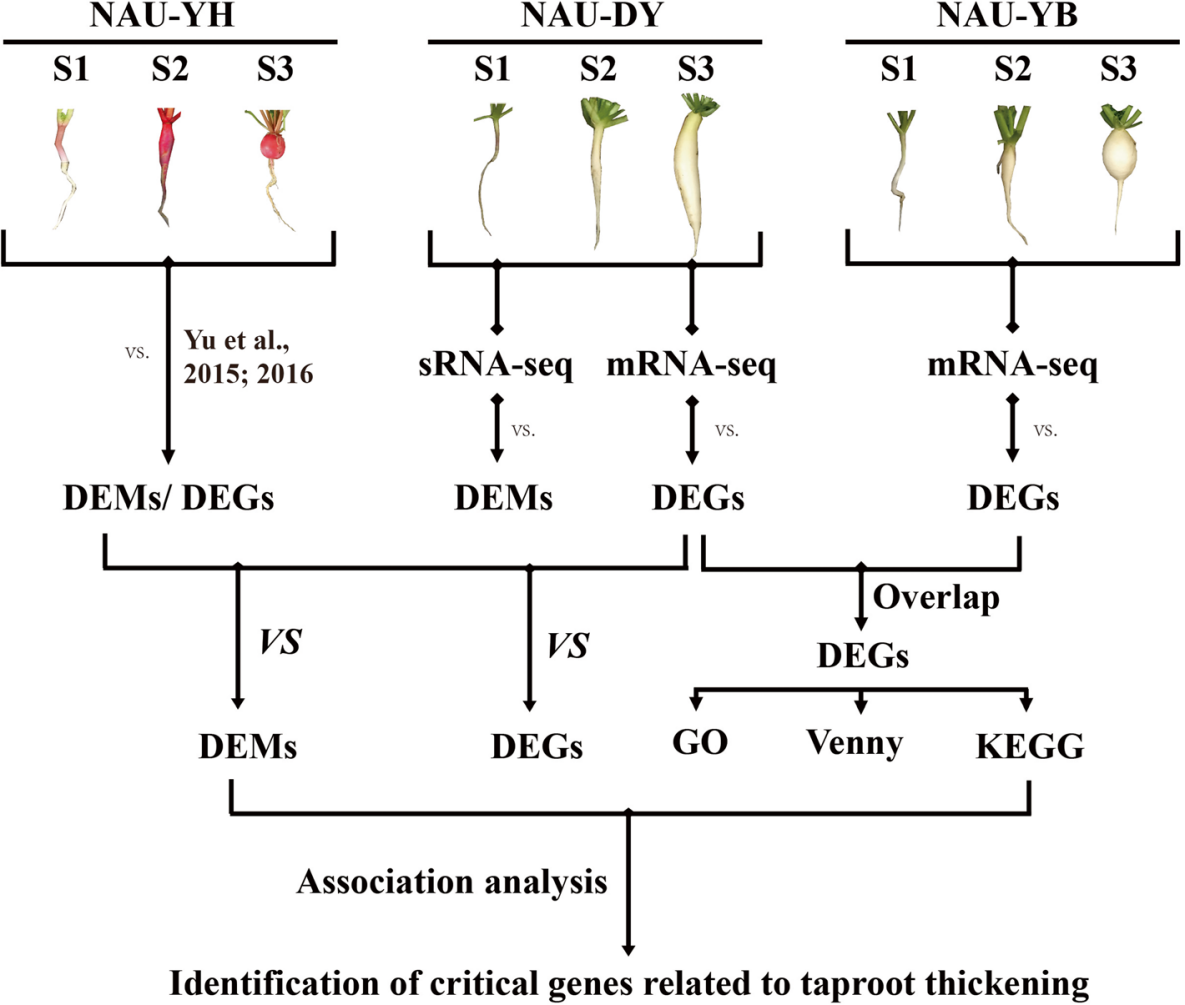
The basic workflow of the integrative transcriptome and miRNA experiment in radish

Small RNA library was generated from total RNA according to the instruction of NEBNext® Multiplex Small RNA Library Prep Set for Illumina® (NEB, USA.) in ‘NAU-DY’. The detailed experimental operation procedures of sRNA library construction and sequencing followed the previous method [22], and the corresponding sRNA-seq libraries were named as DS1, DS2, and DS3 library, respectively. Among these, mRNA-seq and sRNA-seq data of ‘NAU-YH’ were cited from our previous studies [Sequence Read Archive (SRA) with the GenBank accession No.: SRX707630] [13, 17]. The technical workflow of the integrated mRNA-seq and sRNA-seq analysis was shown in Fig. 2.

### Genome mapping and differential expression analysis

Reference genome sequences were downloaded from the radish genome website (ftp://ftp.kazusa.or.jp/pub/radish), and two transcriptome sequences of radish from ‘NAU-YH’ and ‘CKA’ were download from our two published papers, and all these sequences were acted as reference sequences in this study [23, 24]. Prior to the alignment of RNA-seq reads to reference sequences, the raw reads were screened to achieve high quality clean reads by removing adaptor reads and contaminants. For mRNA-seq data analysis, the clean reads were mapped to reference sequences of radish with no more than two mismatchs using TopHat2 software [25]. The FPKM method was used to calculate gene expression level. Read counts data were normalized using TMM method, and *p*-value was calculated with Poisson distribution model. The FDR (false discovery rate) is determined by *p*-value ranges in multiple tests. In this study, the threshold of |log_2_FC (fold change)| > 1 with *q*-value < 0.005 was selected as simulated biological variation to determine whether a gene is significantly differential expression in the DEGseq analysis [26, 27]

For sRNA-seq data analysis, the unique small RNAs were mapped to radish genomic sequence by Bowtie software [28]. Sequences matching non-coding RNAs included rRNAs, tRNAs, snRNAs, and snoRNAs were removed. The remaining unique sequences were searched against with known miRNA sequence by miRBase 21 software for known miRNA identification. Then the remaining unknown sRNAs was used to predict novel miRNAs by miREvo [29] and mirdeep2 software [30]. Differentially expressed miRNAs (DEMs) from different developmental stages of taproot thickening were identified using DESeq software [31]. For all comparisons, miRNAs with |log_2_ FC | > 1 and an adjusted *q*-value < 0.01 were assigned as DEMs.

### miRNA target prediction and annotation

Prediction of miRNA target gene was performed by psRobot_tar in psRobot [32]. GO classification (GO database, http://www.geneontology.org.) and KEGG pathway (KEGG database, http://www.genome.jp/kegg/) methods were carried out for allocating genes to different functional categories and predicting their biological functions, respectively. The KEGG pathway enrichment and GO enrichment analysis were performed with the condition of corrected *p* value < 0.05.

### Reverse transcription quantitative PCR (RT-qPCR) analysis

Total RNA and miRNA extraction (Tiangen) and reverse transcription (Takara) were conducted according to the manufacturer’s instructions. RT-qPCR was performed using a SYBR Primix Ex Taq kit (TaKaRa), and the amplification reactions were conducted on ROCHE LightCycler 480 instruments [33]. The *RsActin* and 5.8S ribosomal RNA (rRNA) were used as the reference genes for normalization, respectively. The relative expression level of each gene was calculated by 2^-△△*C*T^ method. Three replicates and Duncan’s test (*P* < 0.05) were conducted, and the Pearson correlation coefficient was calculated by DPS software to evaluate the correlation of gene expression patterns from RNA-Seq and RT-qPCR. Primers were designed by Beacon Designer 7.0 (Additional file 1: Table S1).

## Results

### Analysis of sRNA and mRNA sequencing data

A total of 14.81 M, 15.65 M, and 16.95 M clean reads were obtained from three development stages during taproot thickening in ‘NAU-DY’. For the length of 18 to 30 nt reads, a total of 10.86 M, 12.20 M, and 13.06 M clean reads were generated from DS1, DS2, and DS3 library, respectively. All the three libraries in length distribution showed a similar size characteristic ranging from 18 to 30 nt, especially for the majority of sRNA reads enriched at the length of 21 and 24 nt (Additional file 1: Figure S1). Among them, 7.56 M (69.59%) sRNA, 9.58 M (78.52%) sRNA, and 10.55 M (80.79%) sRNA reads were successfully mapped to reference sequences corresponding to DS1, DS2, and DS3 library, respectively (Additional file 1: Table S2).

To identify regulated genes during taproot thickening in radish, six cDNA libraries from two advanced inbred lines were constructed from plants at three developmental stages of taproot thickening (S1, S2, and S3). In the line of ‘NAU-DY’, a total of 34.84 M, 25.42 M, and 28.28 M clean reads were obtained from DY_S1, DY_S2, and DY_S3, respectively, from which 22.62 M (64.91%), 16.19 M (63.69%), and 18.10 M (64.01%) were correspondingly uniquely mapped (Additional file 1: Table S3). While in the line of ‘NAU-YB’, totally 32.54 M, 33.10 M, and 31.62 M clean reads were generated from YB_S1, YB_S2 and YB_S3, respectively, among which 21.14 M (64.95%), 22.36 M (67.56%), and 21.21 M (67.08%) were uniquely mapped from YB_S1, YB_S2 and YB_S3, respectively (Additional file 1: Table S3).

### DEMs identification during three developmental stages of taproot thickening

**Fig. 3:**
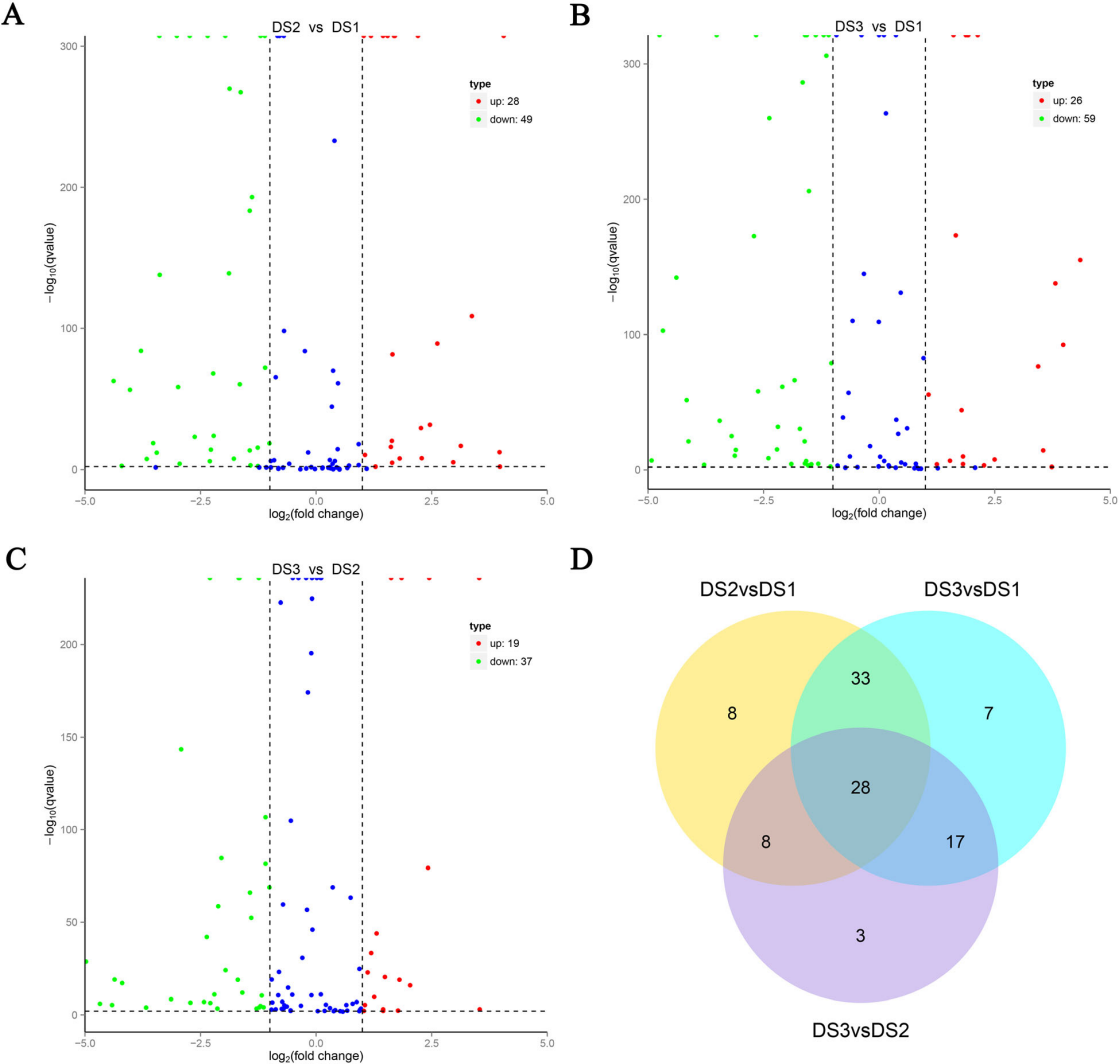
Identification of DEMs during radish taproot thickening. **a**. Volcano plot of miRNAs; **b**. Venny chart of miRNAs

A total of 77, 85, and 56 DEMs were identified from DS2 vs DS1, DS3 vs DS1, and DS3 vs DS2, respectively (Fig. 3a, b, c and Additional file 1: Table S4). In detail, compared with pre-cortex splitting stage, 28 up-regulated miRNAs and 49 down-regulated miRNAs were identified at cortex splitting stage. Meanwhile, 85 DEMs including 26 up-regulated and 59 down-regulated miRNAs were identified in DS3 vs DS1. However, only 56 miRNAs were identified to be differentially expressed in DS3 vs DS2, with 19 up- and 37 down-regulated miRNAs in radish. Interestingly, only eight, seven and three miRNAs were specifically expressed in DS2 vs DS1, DS3 vs DS1, and DS3 vs DS2, respectively; whereas 33, 17 and eight DEMs were shared with each pairwise comparison, respectively; but 28 DEMs were shared among three comparisons (Fig. 3d). Heatmap clustering of DEMs was shown in Additional file 1: Figure S1, and the results indicated that the expression levels of those miRNAs exhibited characteristics of dynamic change during radish taproot thickening.

A total of 16 DEMs were shared between ‘NAU-YH’ and ‘NAU-DY’ from sRNA-seq data (Table 1). Among these DEMs, 14 DEMs were conserved miRNAs, and two DEMs were non-conserved miRNAs. Interestingly, the expression patterns of miR394a, miR408-5p, and miR828 were similar between ‘NAU-YH’ and ‘NAU-DY’; whereas that of miR395a was changed between ‘NAU-YH’ and ‘NAU-DY’ in two lines of three corresponding comparisons (Table 1). Meanwhile, compared with the small-size genotype ‘NAU-YH’, a total of 12 DEMs were specifically differential expressed during taproot thickening in large-size radish genotype ‘NAU-DY’ (Table 2). Among these, miR165a-3p and miR165a-5p were down-regulated in DS3 vs DS1 and DS3 vs DS2 pairs, and miR167c-5p and miR167d were down-regulated in DS2 vs DS1 and DS3 vs DS1 pairs; whereas miR167a-3p were up-regulated in each comparison pairs. These results suggested that miR394a, miR408-5p, and miR828 might be involved in taproot thickening, while miR395a and DEMs specific to ‘NAU-DY’ might contribute to root-type differences.

**Table 1:** Overview of DEMs shared between ‘NAU-YH’ and ‘NAU-DY’

DEMs	NAU-YH			NAU-DY		
	log_**2**_ (S2/S1)	log_**2**_ (S3/S1)	log_**2**_ (S3/S2)	log_**2**_ (S2/S1)	log_**2**_ (S3/S1)	log_**2**_ (S3/S2)
miR156a-3p	9.52	N	−9.52	−5.3591	− 9.405	− 4.3581
miR156c-3p	−13.12	− 13.12	N	− 3.3905	− 5.0363	−1.9579
miR157a-3p	N	−1.72	− 2.44	− 2.2267	− 6.8916	− 4.977
miR171b-3p	10.26	N	−10.26	−2.627	− 3.4505	− 1.1357
miR171b-5p	−9.86	−9.86	N	−2.3005	−5.8566	−3.6757
miR172c	3.37	2.74	N	5.5808	8.3388	2.4459
miR172e-3p	−4.22	−4.28	N	6.0129	7.5173	1.1922
miR394a	7.73	7.25	N	1.6853	1.9103	N
miR395a	−7.57	−7.57	N	3.3711	3.9826	N
miR395b	7.41	6.36	−1.05	2.4611	1.8126	N
miR397a	−3.92	−6.29	−2.38	−5.7315	−4.3851	1.0342
miR408-5p	−2.01	− 4.93	− 2.91	−1.9648	−2.6612	− 1.0086
miR824-3p	−3.36	−3.53	N	−1.6303	N	1.3092
miR828	−1.80	−1.66	N	−6.2059	−5.7862	N
miR400	−1.19	−1.86	N	−1.2587	N	N
miR858a	N	1.38	1.19	−3.3814	−1.035	2.0342

**Table 2:** Identification of DEMs specific to ‘NAU-DY’ with large acicular root

sRNA	DS2 reads	DS1 reads	log_**2**_FC	DS3 reads	DS1 reads	Log_**2**_FC	DS3 reads	DS2 reads	log_**2**_FC
miR165a-3p	N	N	N	4219	12,833	−2	3692	11,787	−2
miR165a-5p	N	N	N	83	295	−2	73	315	−2
miR167a-3p	63	13	2	113	10	4	1646	760	1
miR167c-3p	39	183	−2	1881	546	2	N	N	N
miR167c-5p	42	332	−3	42	137	−2	N	N	N
miR167d	677	1452	−1	58	248	−2	N	N	N
miR319a	162,378	373,420	−1	N	N	N	475,335	131,886	2
miR5658	N	N	N	7	0	4	6	0	4
miR8175	N	N	N	407	141	2	356	102	2
miR857	3	199	−6	8	149	−4	7	2	2
miR170-5p	0	57	−7	0	43	−6	N	N	N
miR393a-5p	N	N	N	5	14	−1	N	N	N

### DEGs identification and functional enrichment analysis

A total of 4131, 4979, and 1635 genes were differentially expressed in YB_S2 vs YB_S1, YB_S3 vs YB_S1, and YB_S3 vs YB_S2, respectively. Meanwhile, 2499, 3970, and 1924 DEGs were identified from DY_S2 vs DY_S1, DY_S3 vs DY_S1, and DY_S3 vs DY_S2, respectively. Of these DEGs, a total of 2606 DEGs were shared between ‘NAU-YB’ and ‘NAU-DY’ (Additional file 2: Table S5). Heatmaps of DEGs indicated that the expression patterns of DEGs were similar between ‘NAU-YB’ and ‘NAU-DY’ (Additional file 1: Figure S2); whereas more DEGs existed and their expression patterns were more various in ‘NAU-YH’ than those in ‘NAU-YB’ and ‘NAU-DY’ [13].

GO enrichment analysis was performed among 2606 DEGs that shared according to the same comparison pairs from ‘NAU-YB’ and ‘NAU-DY’. ‘cellulose microfibril organization’ (GO:0010215), ‘cell growth’ (GO:0016049), ‘carbohydrate biosynthetic process’ (GO: 0016051), and ‘S-adenosylmethionine biosynthetic process’ (GO:0006556) were specifically enriched in S3 vs S1, whereas ‘carbohydrate metabolic process’ (GO: 0005975) was shared with each comparison pairs in taproot thickening of radish (Table 3). Meanwhile, KEGG enrichment analysis was used to identify the critical pathway that genes involved in taproot thickening. ‘starch and sucrose metabolism’ (ath00500) was shared among three comparison pairs, ‘phenylpropanoid biosynthesis’ (ath00940), ‘glucosinolate biosynthesis’ (ath00966) was shared between two comparison pairs (S2 vs S1 and S3 vs S1), whereas ‘thiamine metabolism’ (ath00730) was specifically enriched in S3 vs S2 (Table 4). The results suggested that it was a process of substances and energy metabolism, which promotes cell growth and organ enlargement during taproot thickening in radish.

**Table 3:** GO significantly enrichment of DEGs shared between ‘NAU-DY’ and ‘NAU-YB’

GO_accession	Description	Corrected_***p-***Value	DEG number
S2 VS S1			
GO:0005975	carbohydrate metabolic process	0.0036482	32
S3 VS S1			
GO:0010215	cellulose microfibril organization	0.024918	5
GO:0016049	cell growth	0.024918	5
GO:0016051	carbohydrate biosynthetic process	0.024918	29
GO:0006556	S-adenosylmethionine biosynthetic process	0.024918	4
S2 VS S1-S3 VS S1			
GO:0005975	carbohydrate metabolic process	1.40E-06	72
GO:0055114	oxidation-reduction process	2.49E-06	90
GO:0044283	small molecule biosynthetic process	5.82E-06	34
S2 VS S1-S3 VS S2			
GO:0005975	carbohydrate metabolic process	4.67E-09	22
GO:0008152	metabolic process	0.0048322	56
S2 VS S1-S3 VS S1-S3 VS S2			
GO:0005975	carbohydrate metabolic process	0.010675	11

**Table 4:** KEGG significantly enrichment of DEGs shared between ‘NAU-DY’ and ‘NAU-YB’

#Term	ID	Input number	Corrected ***P***-Value
**S3 VS S1**			
Phenylalanine metabolism	ath00360	16	0.049828146
Cysteine and methionine metabolism	ath00270	14	0.051167179
Phenylpropanoid biosynthesis	ath00940	18	0.0527419
Ubiquinone and other terpenoid-quinone biosynthesis	ath00130	6	0.218324112
ABC transporters	ath02010	5	0.223834934
Biosynthesis of secondary metabolites	ath01110	64	0.414801288
Isoquinoline alkaloid biosynthesis	ath00950	4	0.414801288
Cutin, suberine and wax biosynthesis	ath00073	4	0.414801288
**S3 VS S2**			
Thiamine metabolism	ath00730	3	0.020228102
**S2 VS S1-S3 VS S1**			
Glucosinolate biosynthesis	ath00966	13	3.79E-07
Starch and sucrose metabolism	ath00500	27	0.001822699
2-Oxocarboxylic acid metabolism	ath01210	15	0.002400877
Biosynthesis of secondary metabolites	ath01110	87	0.002498601
Phenylpropanoid biosynthesis	ath00940	22	0.003925529
Phenylalanine metabolism	ath00360	18	0.004088511
Sulfur metabolism	ath00920	10	0.004445186
Selenocompound metabolism	ath00450	5	0.067434074
Tryptophan metabolism	ath00380	8	0.067434074
Cysteine and methionine metabolism	ath00270	13	0.067434074
Nitrogen metabolism	ath00910	7	0.126626602
Phenylalanine, tyrosine and tryptophan biosynthesis	ath00400	8	0.159801465
Glycine, serine and threonine metabolism	ath00260	9	0.159801465
Glutathione metabolism	ath00480	11	0.159801465
Tyrosine metabolism	ath00350	6	0.215571217
Flavonoid biosynthesis	ath00941	4	0.239223498
**S2 VS S1-S3 VS S2**			
Fructose and mannose metabolism	ath00051	5	0.007626905
Amino sugar and nucleotide sugar metabolism	ath00520	7	0.007626905
Starch and sucrose metabolism	ath00500	8	0.013150643
**S3 VS S2-S3 VS S1**			
Flavonoid biosynthesis	ath00941	4	0.035061213
**S2 VS S1-S3 VS S1-S3 VS S2**			
alpha-Linolenic acid metabolism	ath00592	3	0.005396308
Starch and sucrose metabolism	ath00500	5	0.006062768

### DEM target prediction and association analysis of DEGs and DEMs

A total of 1280 targets were predicted with psRobot software from sRNA data of ‘NAU-DY’ (Additional file 1: Table S6). Among these targets, totally 849 and 431 targets belonged to 68 known miRNAs and 36 novel miRNAs, respectively (Additional file 1: Table S6). Of these, even though these miRNAs targeted different genes, the annotations of several targets belonged to the same families, for example, several known miRNAs including miR156a-3p, miR159b-3p, miR159c, miR5658, miR827, miR828, miR858a, miR858b, and novel miRNAs including novel_119, novel_136, novel_1, novel_37, and novel_96 targeted *Myb* domain genes (Additional file 1: Table S6).

A total of 12 DEMs were specifically differential expressed during taproot thickening in large-size radish genotype ‘NAU-DY’ when compared with the small-size genotype ‘NAU-YH’ (Table 2). To explore miRNA-mRNA regulatory network in taproot thickening, the association analysis between miRNA and mRNA of 12 DEMs that specific to ‘NAU-DY’ was performed. The results showed that miR167c-5p (targeted by *ARF8*), miR393a-5p (targeted by *bHLH77*), miR5658 (targeted by *APL*) were identified to be specifically differentially expressed during radish taproot thickening of ‘NAU-DY’ (Additional file 1: Table S6).

### RT-qPCR validation

**Fig. 4:**
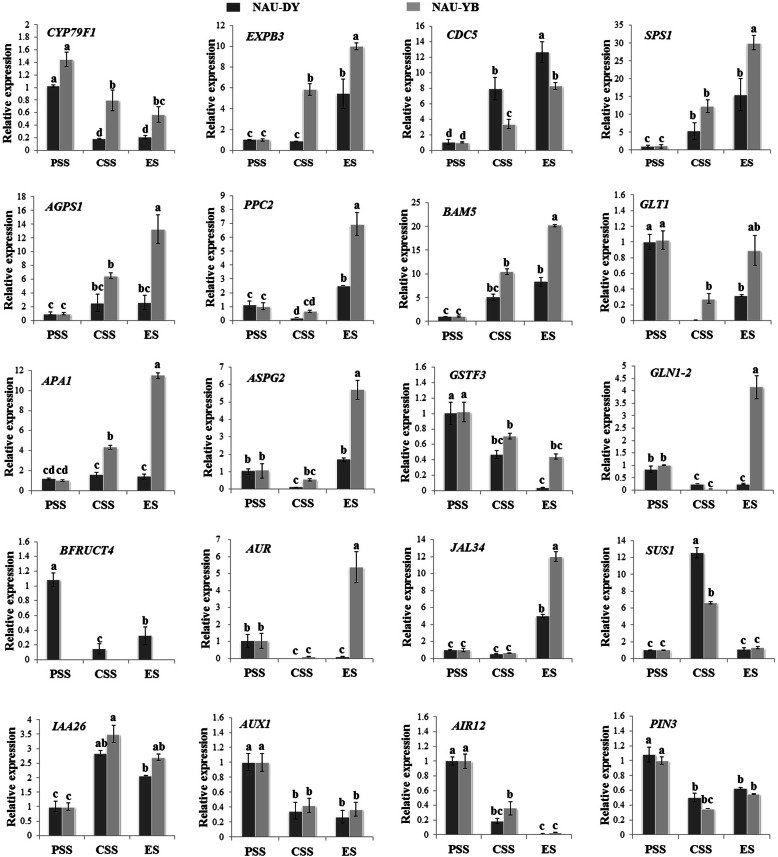
The validation of expression levels of selected DEGs related to radish taproot thickening

To explore genes expression patterns during radish taproot thickening, 20 genes were performed for RT-qPCR analysis from RNA-seq data of ‘NAU-DY’ and ‘NAU-YB’. RT-qPCR results showed that the expression levels of auxin-induced in root cultures protein 12 (*RsAIR12*), auxin transporter protein 1 (*RsAUX1*), auxin efflux carrier component 3 (*RsPIN3*), dihomomethionine N-hydroxylase (*CYP79F1*), glutamate synthase 1 (*GLT1*), glutathione S-transferase F3 (*GSTF3*), and acid beta-fructofuranosidase 4 (*BFRUCT4*) were higher at PSS than that at CSS and ES, whereas the expression level of auxin-responsive protein (*IAA26*) and sucrose synthase 1 (*SUS1*) were higher at CSS than that at PSS and ES (Fig. 4). Meanwhile, expansin B3 (*EXPB3*), cell division cycle 5-like protein (*CDC5*), sucrose-phosphate synthase 1 (*SPS1*), jacalin-related lectin 34 (*JAL34*), glutamine synthetase cytosolic isozyme 1–2 (*JLN1–2*), aspartic protease in guard cell 2 (*ASPG2*), aspartic proteinase A1 (*APA1*), beta-amylase 5 (*BAM5*), phosphoenolpyruvate carboxylase 2 (*PPC2*), and glucose-1-phosphate adenylyltransferase (*AGPS1*) were higher at ES than that at CSS and PSS (Fig. 4). In addition, RT-qPCR expression profiles showed that 18 of 20 randomly selected genes were in agreement with the RNA-Seq data, and two genes including *JAL34* and *AUR* showed difference only at ES and CSS, respectively. Overall, the Pearson correlation coefficient results displayed a positive correlation between RNA-seq data and RT-qPCR analysis at the mRNA level (*r* = 0.78, *p* = 0.0006), indicating the reliability of the transcriptomic data (Additional file 1: Figure S3).

**Fig. 5:**
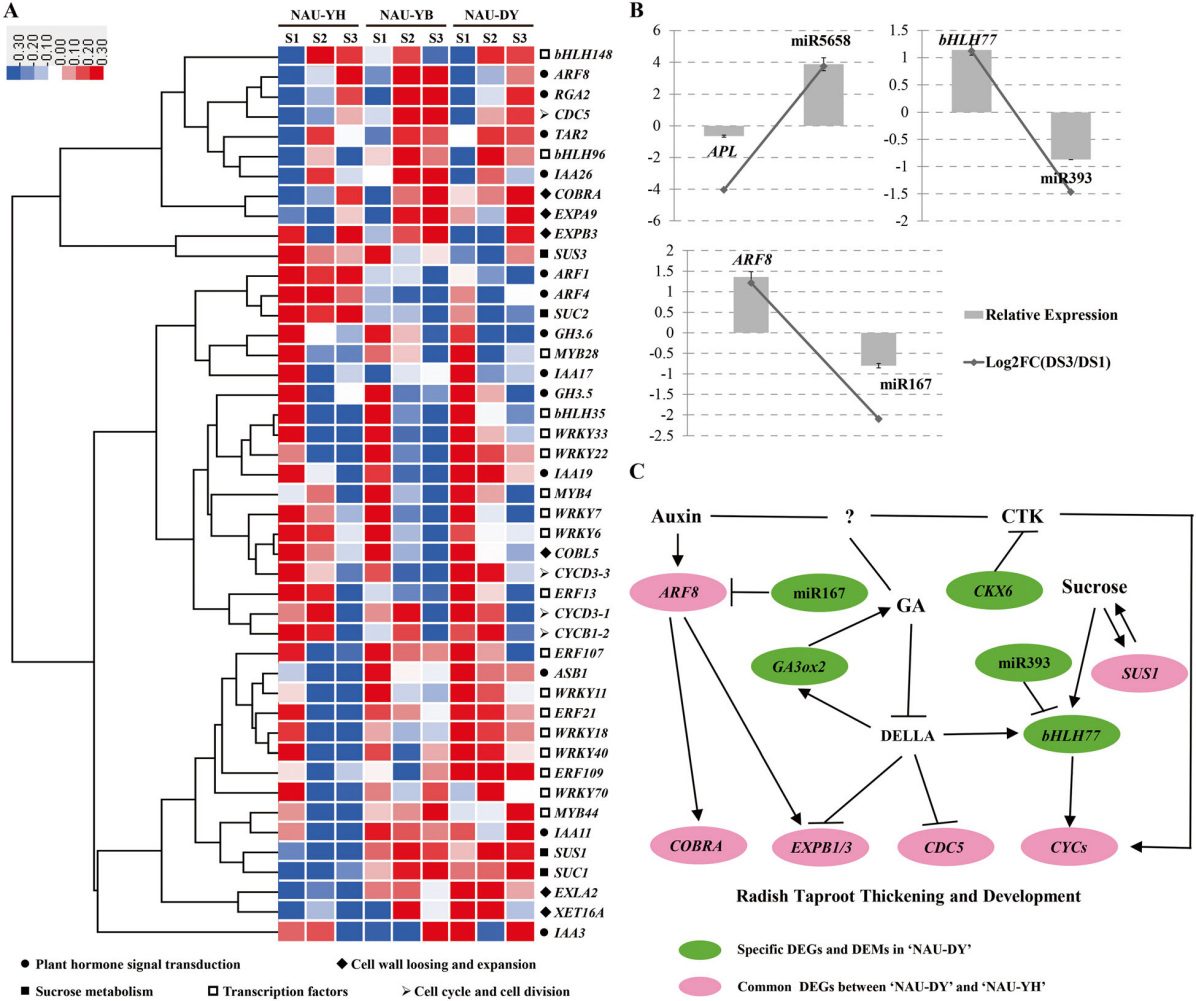
A putative regulatory model of radish taproot thickening and development. **a**. Heatmap of shared DEGs among three advance inbred lines; **b**. The expression profile of DEMs and their corresponding target genes; **c**. A proposed regulatory model of radish taproot thickening and development

To verify the expression patterns of miRNAs and their corresponding targets in radish, a total of 14 DEMs and three differentially expressed target genes were selected for RT-qPCR analysis. As shown in Figure S4**,** the expression patterns of RT-qPCR analysis were in line with that obtained from sRNA-seq, whereas they had differences on the degrees of differential expression between the two methods. This inconsistency might be caused by two different calculation methods. Moreover, the negative correlations between miRNAs and their corresponding targets (miR167-*ARF8*, miR393-*bHLH77*, miR5658-*APL*) could be found at the expression levels, suggesting the reliability of the sRNA-seq data and miRNA-mediated gene silencing involved in radish taproot thickening (Fig. 5).

### Molecular dynamic regulation network of taproot formation and development

Radish taproot thickening has a complex molecular regulation mechanism [13, 15, 17]. In general, the substances involved in morphogenesis including sucrose, starch, and protein are indispensable for taproot formation and development in radish, and a series of genes (e.g. *SUS1*, *SUS3*, *SUC1*, *SUC2*) were involved in these processes. Among them, *SUS1* might contribute to taproot thickening in radish with long and thick root [15]. Genes involved in plant hormone biosynthesis and signal transduction including auxin (e.g. *SAUR40*, *IAA26*, *IAA3*, *IAA7*), CTK (e.g. *LOG8*, *CKX6*), and GA (e.g. *GA2ox1*, *GA3ox2*, *RGA2*) are likely to be important regulators in signal transduction process for radish taproot thickening [34, 35]. Cell cycle is regulated by F-box protein (e.g. *CYCU4–1*, *CYCD3–1*, *CDC5*) and cyclin-dependent kinase (e.g. *KRP5*) [36–38], which might determine cell number of radish taproot thickening. Cell expansion is regulated by expansin proteins (e.g. *EXPA9*, *EXPB1/3*), xyloglucan endotransglucosylase/ hydrolase protein (e.g. *XTH9*, *XTH7*), and root cell elongation factor (e.g. *COBRA*) [35, 39], which might determine cell size during taproot thickening. In addition, several transcription factors (TFs) were found to be involved in root development in other plants including rice, and *Arabidopsis*, and some TFs including bHLHs (e.g. miR393-*bHLH77*, *bHLH96*, *bHLH148*), MYBs (*MYB4*, *MYB28*, *MYB44*), and WRKYs (e.g. *WRKY12*, *WRKY19*, *WRKY33*) were identified in this study, indicating that they may play critical roles in taproot thickening in radish (Additional file 2: Table S5) [40–43].

Totally, 45 shared genes were differentially expressed in three advanced inbred lines with different root size, and their corresponding expression patterns were shown in Fig. 5a. The results indicated that most DEGs shared the similar expression patterns, whereas several DEGs (e.g. *SUS1*, *SUC1*, *EXPB3*, *EXLA2*, and *ERF109*) exhibited differential expression patterns. Interestingly, gibberellin 3 oxidase 2 (*GA3ox2*)*,* cytokinin oxidase 6 (*CKX6*), miR167c-5p (targeted by *ARF8*), and miR393a-5p (targeted by *bHLH77*) were specifically differentially expressed in ‘NAU-DY’. Based on these results, a putative regulatory model of radish taproot thickening and development was put forward (Fig. 5b, c). In short, sucrose (the key gene of sucrose metabolism: *SUS1*) as a signal molecular could induce miR393-*bHLH77* specific expression [44, 45], and then regulation of downstream genes (*CYCs*) played important roles in cell division during radish taproot thickening [40, 43, 46]. Some genes involved in the biosynthesis and signal transduction of auxin (miR167-*ARF8*), CTK (*CKX6*) and GA (*GA3ox2*) also regulated several functional genes (*COBRA*, *EXPB1/3*, *CDC5*), indicating that they might play roles in initiating radish taproot thickening and development (Fig. 5) [47].

## Discussion

Integrative mRNA-seq and sRNA-seq approach provided valuable tool for exploiting the potential critical genes and uncovering complex regulatory networks for the traits [48–50]. Radish taproot thickening is a complex biological process that consisted of a series of material accumulation and signal transduction pathway. To date, the molecular mechanism of taproot formation was still not fully uncovered in radish. In this study, an integrated mRNA-seq and sRNA-seq analysis was performed during the taproot thickening in three radish advanced inbred lines to further understanding the molecular mechanism underlying the taproot formation. As size varied in fleshy root, three representative radish advanced inbred lines were used in this study. A total of 2606 DEGs were shared between ‘NAU-DY’ and ‘NAU-YB’, whereas 16 DEMs were shared between ‘NAU-YH’ and ‘NAU-DY’ and 12 DEMs were specifically differentially expressed in ‘NAU-DY’. Moreover, several critical genes including *SUS1*, *EXPB3*, and *CDC5* were characterized and profiled by RT-qPCR analysis. This study represents a systematical report on characterization of the potential critical genes involved in taproot formation by integrative mRNA-seq and sRNA-seq approach in three radish advanced lines.

### Critical genes related to root - type differences

Genes related to root-type difference are considered to be candidates that promote or repress taproot thickening in radish [51]. A total of 140 and 70 specifically expressed genes were identified in skinny-type roots and thick-type roots using suppression subtractive hybridization (SSH), respectively [18]. Several genes involved in phenylpropanoid metabolism were overexpressed in the skinny-root-type cultivar, and phenylpropanoid was the precursor in lignin synthesis, suggesting lignin biosynthetic pathway was involved in radish taproot thickening [52]. Furthermore, genes related to ethylene production and root hair elongation were also overexpressed in skinny-type roots, and the reason may be that ethylene and lateral root development inhibit cell expansion and elongation of main root [53]. Therefore, the DEGs between skinny- and thick-root cultivars were probably involved in root-type variation.

In this study, a total of 12 DEMs belonging to miR165, miR167, miR319, miR5658, miR8175, miR857, miR170, and miR393 family miRNAs were specifically differentially expressed during taproot thickening in large acicular radish ‘NAU-DY’, which might be related to root-type variation. On the other hand, many DEGs were identified to be specifically or commonly expressed between ‘NAU-YH’ and ‘NAU-DY’ (Fig. 5). For example, genes involved in starch and sucrose metabolism including sucrose synthase 4 (*SUS4*), sucrose-phosphate synthase 2 (*SPS2*), and 6-phosphofructokinase (*FK1*, *FK7*) were specifically expressed in ‘NAU-DY’, whereas *SUS1*, sucrose synthase 3 (*SUS3*), sucrose transport protein (*SUC1*, *SUC2*), sucrose-phosphate synthase 2 (*SPS1*), and 6-phosphofructokinase (*FK3*, *FK6*) were co-expressed in ‘NAU-DY’ and ‘NAU-YH’. Association analysis of mRNA and sRNA results showed that miR167c-5p (targeted by *ARF8*), miR393a-5p (targeted by *bHLH77*), miR5658 (targeted by *APL*) were identified to be significantly differentially expressed during radish taproot thickening of ‘NAU-DY’, indicating that miR393-*bHLH77*, miR167-*ARF8*, and miR5658-*APL* (a member of MYB) might play critical roles in taproot thickening of radish.

### SUS1 and CDC5 might contribute to taproot thickening in radish

Carbohydrate metabolism was prominently activated in thickening roots, particularly in cell proliferating tissues, among which the expression level of *SUS1* was associated with root thickening rates [15]. In this study, the GO term ‘carbohydrate metabolism process’ (GO: 0005975) was significantly enriched and shared by any comparison pair for taproot thickening of radish, irrespective of the size of the root, which was in agreement with a previous report in radish [13, 15], suggesting that carbohydrate metabolism would probably be vital for taproot thickening.

Interestingly, among these genes involved in carbohydrate metabolism process, *SUS1* gene showed differential expression patterns among three advanced inbred lines of radish. For large acicular radish, the expression level of *SUS1* (Rsa1.0_00483.1_g00003.1) reached peak at cortex splitting stage and much higher than those in medium obovate and small circular radish (FD536105). Furthermore, *SUS gene* was of importance on the development of potato tuberization [54], tomato fruits setting [55], carrot root formation [56], maize grain formation [57], wheat grain formation [58], and rice grain formation [59]. In this study, RT-qPCR validation result indicated that *SUS1* displayed high expression patterns at CSS, particularity in ‘NAU-DY’, indicating *SUS1* might play a vital role on taproot thickening process, particularly for the thickening of large acicular radish rather than the small circular radish (Fig. 4).

*CDC5* is a cell cycle regulator that encoding a MYB-related protein. Recently, increasing reports of *AtCDC5* plays a positive regulation for miRNAs accumulation, which control plant growth and development [60]. Meanwhile, *CDC5* gene was crucial to cell cycle during the G2 period, and the phase transition of G2 to M (G2/M) was affected in the *AtCDC5*-RNAi transformants [37], and *AtCDC5*-VIGS transformants died before bolting and accelerated cell death [38]. Interestingly, *CDC5* was identified to be up-regulated during taproot thickening, whatever RNA-seq or iTRAQ-seq method, which would be play important role in cell division of taproot thickening in radish [13, 35]. In this study, RT-qPCR validation result was approximately consistent with those of previous studies, and *CDC5* showed high abundance expression at ES (Fig. 4). These results preliminarily suggested that *CDC5* might play an important role on the growth and development of radish taproot thickening.

### COBRA might be required for cell elongation of radish taproot

Plant growth and development are promoted by a series of targeted cell division and cell expansion. The plant cell wall provides fundamental mechanical support for the plant body and determinant of cell size and shape. As the main component of cell wall, cellulose microfibril organization is one of determinant of cell expansion. In this study, the GO term ‘cellulose microfibril organization’ (GO: 0010215) was significantly enriched in S3 vs S1 pair comparison between ‘NAU-DY’ and ‘NAU-YB’, and totally two *COBRA* and three *COBRA-like* genes including *COBL2*, *COBL5* and *COBL8* were identified to be differentially expressed (Additional file 2). Previous studies showed that *COBRA* involved in the cellulose synthesis, controlling the content of cellulose in plant cell wall and the function of cell directional elongation [61–64]. Interestingly, in this study, all *COBRA* genes were up-regulated, whereas all *COBRA-like* genes were down-regulated in S3 vs S1 pair comparison between ‘NAU-DY’ and ‘NAU-YB’, suggesting that *COBRA* might play a critical role on cell elongation for taproot thickening in radish.

## Conclusions

This is the first report on integrative analysis of transcriptome and miRNA in three radish advanced inbred lines during taproot thickening. A total of 2606 DEGs were shared between ‘NAU-DY’ and ‘NAU-YB’, which significantly enriched in ‘phenylpropanoid biosynthesis’, ‘glucosinolate biosynthesis’, and ‘starch and sucrose metabolism’ pathway. Meanwhile, a total of 16 DEMs were shared between ‘NAU-DY’ and ‘NAU-YH’, whereas 12 miRNAs showed specifically differential expression during taproot thickening in ‘NAU-DY’ with large acicular root when compared with ‘NAU-YH’ with small circular root. Association analysis between DEMs and DEGs indicated that miR393-*bHLH77*, miR167-*ARF8*, and miR5658-*APL* might be related to root-type variation in radish. Furthermore, RT-qPCR validation results indicated that the DEGs/ DEMs evaluated were highly in agreement with the RNA-Seq data. These finding would provide valuable information on comprehensive understanding of the molecular regulatory mechanism underlying taproot thickening in radish, and facilitate further genetic manipulation and quality improvement of root vegetable crops.

## Supplementary information

**Additional file 1 Figure S1**. The sequence length distribution of small RNAs during radish taproot thickening. **Figure S2**. The heatmap of DEGs and DEMs in radish taproot thickening. a. Heatmap of DEMs in ‘NAU-DY’; b, c represents heatmap of DEGs in ‘NAU-DY’ and ‘NAU-YB’, respectively. **Figure S3**. Comparative analysis of gene expression profiles from RNA-seq and RT-qPCR. 20 randomly selected genes by RNA-seq and RT-qPCR showing different expression patterns in three comparative groups (DS2 vs DS1, DS3 vs DS1, and DS3 vs DS2) during taproot thickening. Each data point represents the log_2_ normalized expression level obtained from RNA-seq (x axis) and RT-qPCR (y axis) analyses. **Figure S4**. RT-qPCR validation of 14 DEMs during radish taproot thickening. The relative expression of DEMs between DS2 and DS1 libraries (a), DS3 and DS1 libraries (b) and DS3 and DS2 libraries (c) were analyzed by the 2^−ΔΔ*C*T^ method. **Table S1**. Primer sequences for RT-qPCR assay. **Table S2**. Summary of small RNA sequencing data. **Table S3**. Summary of mRNA sequencing data. **Table S4**. Detailed information of DEMs during radish taproot thickening in ‘NAU-DY’. **Table S6**. DEMs and their corresponding targets extracted from RNA-seq.

**Additional file 2 Table S5**. Overview of DEGs shared between ‘NAU-DY’ and ‘NAU-YB’.

## Data Availability

All the raw data supporting the results of this article have been deposited into the NCBI Sequence Read Archive, which included six cDNA libraries (for mRNA-seq) and three small RNA libraries of the raw sequencing data under accession number SRP158483 (https://www.ncbi.nlm.nih.gov/sra/?term=SRP158483). The processed data supporting the results of this article are included within the article and supplementary profiles.

## Abbreviations

FDR: False discovery rate
DEGs: Differentially expressed genes
DEMs: Differentially expressed miRNAs
KEGG: Kyoto encyclopedia of genes and genomes
RT-qPCR: Reverse transcription quantitative PCR

## References

[1] Moreno-Risueno MA, Sozzani R, Yardımcı GG, Petricka JJ, Vernoux T, Blilou I (2015). Transcriptional control of tissue formation throughout root development. Science.

[2] Song W, Xue R, Song Y, Bi Y, Liang Z, Meng L (2018). Differential response of first-order lateral root elongation to low potassium involves nitric oxide in two tobacco cultivars. J Plant Growth Reg.

[3] Hochholdinger F, Marcon C, Baldauf JA, Yu P, Frey FP (2018). Proteomics of maize root development. Front Plant Sci.

[4] Zhang Z, Zhang X, Lin Z, Wang J, Xu M, Lai J (2018). The genetic architecture of nodal root number in maize. Plant J.

[5] Sanchez DL, Liu S, Ibrahim R, Blanco M, Lübberstedt T (2018). Genome-wide association studies of doubled haploid exotic introgression lines for root system architecture traits in maize (*Zea mays* L.). Plant Sci.

[6] Zhao Y, Cheng S, Song Y, Huang Y, Zhou S, Liu X (2015). The interaction between rice ERF3 and WOX11 promotes crown root development by regulating gene expression involved in cytokinin signaling. Plant Cell.

[7] Chen H, Ma B, Zhou Y, He SJ, Tang SY, Lu X (2018). E3 ubiquitin ligase SOR1 regulates ethylene response in rice root by modulating stability of aux/IAA protein. Proc Natl Acad Sci U S A.

[8] Han Y, Wan HH, Cheng TR, Wang J, Yang WR, Pan HT (2017). Comparative RNA-seq analysis of transcriptome dynamics during petal development in *Rosa chinensis*. Sci Rep.

[9] Yuan SL, Li R, Chen HF, Zhang CJ, Chen LM, Hao QN (2017). RNA-Seq analysis of nodule development at five different developmental stages of soybean (*Glycine max*) inoculated with *Bradyrhizobium japonicum* strain 113-2. Sci Rep.

[10] Yu K, Xu Q, Da X, Guo F, Ding Y, Deng X (2012). Transcriptome changes during fruit development and ripening of sweet orange (*Citrus sinensis*). BMC Genomics.

[11] Feng C, Chen M, Xu CJ, Bai L, Yin XR, Allan AC (2012). Transcriptomic analysis of Chinese bayberry (*Myrica rubra*) fruit development and ripening using RNA-Seq. BMC Genomics.

[12] Zouari I, Salvioli A, Chialva M, Novero M, Miozzi L, Tenore GC (2014). From root to fruit: RNA-Seq analysis shows that arbuscular mycorrhizal symbiosis may affect tomato fruit metabolism. BMC Genomics.

[13] Yu R, Wang J, Xu L, Wang Y, Wang R, Zhu X (2016). Transcriptome profiling of taproot reveals complex regulatory networks during taproot thickening in radish (*Raphanus sativus* L.). front. Plant Sci.

[14] Kitashiba H, Li F, Hirakawa H, Kawanabe T, Zou ZW, Hasegawa Y (2014). Draft sequences of the radish (*Raphanus sativus* L.) genome. DNA Res.

[15] Mitsui Y, Shimomura M, Komatsu K, Namiki N, Shibata-Hatta M, Imai M (2015). The radish genome and comprehensive gene expression profile of tuberous root formation and development. Sci Rep.

[16] Jeong YM, Kim N, Ahn BO, Oh M, Chung WH, Chung H (2016). Elucidating the triplicated ancestral genome structure of radish based on chromosome-level comparison with the Brassica genomes. Theor Appl Genet.

[17] Yu R, Wang Y, Xu L, Zhu X, Zhang W, Wang R (2015). Transcriptome profiling of root microRNAs reveals novel insights into taproot thickening in radish (*Raphanus sativus* L.). BMC Plant Boil.

[18] Zaki HEM, Yokoi S, Takahata Y (2010). Identification of genes related to root shape in radish (*Raphanus sativus*) using suppression subtractive hybridization. Breed Sci.

[19] Zaki HEM, Takahata Y, Yokoi S (2012). Analysis of the morphological and anatomical characteristics of roots in three radish (*Raphanus sativus*) cultivars that differ in root shape. J Hortic Sci Biotechnol.

[20] Sun X, Xu L, Wang Y, Luo X, Zhu X, Kinuthia KB (2016). Transcriptome-based gene expression profiling identifies differentially expressed genes critical for salt stress response in radish (*Raphanus sativus* L.). Plant Cell Rep.

[21] Wang R, Mei Y, Xu L, Zhu X, Wang Y, Guo J (2018). Genome-wide characterization of differentially expressed genes provides insights into regulatory network of heat stress response in radish (*Raphanus sativus* L.). Funct Integr Genomic.

[22] Xu L, Wang Y, Zhai L, Xu Y, Wang L, Zhu X (2013). Genome-wide identification and characterization of cadmium-responsive microRNAs and their target genes in radish (*Raphanus sativus* L.) roots. J Exp Bot.

[23] Yu RG, Xu L, Zhang W, Wang Y, Luo XB, Wang RH (2016). *De novo* taproot transcriptome sequencing and analysis of major genes involved in sucrose metabolism in radish (*Raphanus sativus* L.). Front Plant Sci.

[24] Wang Y, Pan Y, Liu Z, Zhu XW, Zhai LL, Xu L (2013). *De novo* transcriptome sequencing of radish (*Raphanus sativus* L.) and analysis of major genes involved in glucosinolate metabolism. BMC Genomics.

[25] Kim D, Pertea G, Trapnell C, Pimentel H, Kelley R, Salzberg SLS (2013). TopHat2: accurate alignment of transcriptomes in the presence of insertions, deletions and gene fusions. Genome Biol.

[26] He G, Chen B, Wang X, Li X, Li J, He H (2013). Conservation and divergence of transcriptomic and epigenomic variation in maize hybrids. Genome Biol.

[27] Li AL, Liu DC, Wu J, Zhao XB, Hao M, Geng SF (2014). mRNA and small RNA transcriptomes reveal insights into dynamic homoeolog regulation of allopolyploid heterosis in nascent hexaploid wheat. Plant Cell.

[28] Langmead B, Trapnell C, Pop M, Salzberg SL (2009). Ultrafast and memory-efficient alignment of short DNA sequences to the human genome. Genome Biol.

[29] Wen M, Shen Y, Shi S, Tang T (2012). miREvo: an integrative microRNA evolutionary analysis platform for next-generation sequencing experiments. BMC Bioinformatics.

[30] Friedlander MR, Mackowiak SD, Li N, Chen W, Rajewsky N (2011). miRDeep2 accurately identifies known and hundreds of novel microRNA genes in seven animal clades. Nucleic Acids Res.

[31] Anders S, Huber W (2010). Differential expression analysis for sequence count data. Genome Biol.

[32] Wu HJ, Ma YK, Chen T, Wang M, Wang XJ (2012). PsRobot: a web-based plant small RNA meta-analysis toolbox. Nucleic Acids Res.

[33] Xu Y, Zhu X, Gong Y, Xu L, Wang Y, Liu L (2012). Evaluation of reference genes for gene expression studies in radish (*Raphanus sativus* L.) using quantitative real-time PCR. Biochem Biophys Res Co.

[34] Li XJ, Yang JL, Hao B, Lu YC, Qian ZL, Li Y, et al. Comparative transcriptome and metabolome analyses provide new insights into the molecular mechanisms underlying taproot thickening in *Panax notoginseng*. BMC Plant Bio. 2019;19:451. 10.1186/s12870-019-2067-5 PMC6815444 31655543

[35] Xie Y, Xu L, Wang Y, Fan LX, Chen YL, Tang MJ (2018). Comparative proteomic analysis provides insight into complex regulatory network of taproot formation in radish (*Raphanus sativus* L.). Hortic Res.

[36] Calzada A, Sánchez M, Sánchez E, Bueno A (2000). The stability of the Cdc6 protein is regulated by cyclin-dependent kinase/cyclin B complexes in Saccharomyces cerevisiae. J Biol Chem.

[37] Lin ZQ, Yin KQ, Zhu DL, Chen ZL, Gu HY, Qu LJ (2007). AtCDC5 regulates the G2 to M transition of the cell cycle and is critical for the function of Arabidopsis shoot apical meristem. Cell Res.

[38] Lin ZQ, Yin KQ, Wang XX, Liu MH, Chen ZL, Gu HY (2007). Virus induced gene silencing of AtCDC5 results in accelerated cell death in Arabidopsis leaves. Plant Physiol Biochem.

[39] Ko JH, Kim JH, Jayanty SS, Howe GA, Han KH (2006). Loss of function of COBRA, a determinant of oriented cell expansion, invokes cellular defence responses in *Arabidopsis thaliana*. J Exp Bot.

[40] Srilakshmi M, Rebecca SL (2013). The bHLH transcription factor *SPATULA* regulates root growth by controlling the size of the root meristem. BMC Plant Bio.

[41] Gu M, Zhang J, Li H, Meng D, Li R, Dai X (2017). Maintenance of phosphate homeostasis and root development are coordinately regulated by MYB1, an R2R3-type MYB transcription factor in rice. J Exp Bot.

[42] Ballachanda ND, Athikkattuvalasu SK, Kashchandra GR (2007). WRKY75 transcription factor is a modulator of phosphate acquisition and root development in Arabidopsis. Plant Physiol.

[43] Li TT, Yang SY, Kang XK, Lei W, Qiao K, Zhang DW (2019). The bHLH transcription factor gene *AtUPB1* regulates growth by mediating cell cycle progression in Arabidopsis. Biochem Biophys Res Co.

[44] Rolland F, Baenagonzalez E, Sheen J (2006). Sugar sensing and signaling in plants: conserved and novel mechanisms. Plant Biol.

[45] Liu XJ, An XH, Liu X, Hu DG, Wang XF, You CX (2017). MdSnRK1.1 interacts with MdJAZ18 to regulate sucrose-induced anthocyanin and proanthocyanidin accumulation in apple. J Exp Bot.

[46] Yang XM, Ren YL, Cai Y, Niu M, Feng ZM, Jing RN, et al. Overexpression of *OsbHLH107*, a member of the basic helix-loop-helix transcription factor family, enhances grain size in rice (*Oryza sativa* L.). Rice, 2018;11:41. 10.1186/s12284-018-0237-y PMC6054598 30030651

[47] Hu JH, Israeli A, Ori N, Sun TP (2018). The interaction between DELLA and ARF/IAA mediates crosstalk between gibberellin and auxin signaling to control fruit initiation in tomato. Plant Cell.

[48] Long JM, Liu Z, Wu XM, Fang YN, Jia HH, Xie ZZ (2016). Genome-scale mRNA and small RNA transcriptomic insights into initiation of citrus apomixis. J Exp Bot.

[49] Sun Q, Du X, Cai C, Long L, Zhang S, Qiao P (2016). To be a flower or fruiting branch: insights revealed by mRNA and small RNA transcriptomes from different cotton developmental stages. Sci Rep.

[50] Han X, Yin H, Song X, Zhang Y, Liu M, Sang J (2016). Integration of small RNAs, degradome and transcriptome sequencing in hyperaccumulator Sedum alfredii uncovers a complex regulatory network and provides insights into cadmium phytoremediation. Plant Biotechnol J.

[51] Mitsui Y, Nishio T, Kitashiba H (2017). Gene expression profiles during tuberous root development. The Radish Genome.

[52] Vogt T (2010). Phenylpropanoid biosynthesis. Mol Plant.

[53] Kieber JJ, Rothenberg M, Roman G, Feldmann KA, Ecker JR (1993). CTR1, a negative regulator of the ethylene response pathway in Arabidopsis, encodes a member of the Raf family of protein kinases. Cell.

[54] Baroja-Fernández E, Muñoz FJ, Montero M, Etxeberria E, Sesma MT, Ovecka M (2009). Enhancing sucrose synthase activity in transgenic potato (*Solanum tuberosum* L.) tubers results in increased levels of starch, ADPglucose and UDPglucose and total yield. Plant Cell Physiol.

[55] D’Aoust MA, Yelle S, Quoc BN (1999). Antisense inhibition of tomato fruit sucrose synthase decreases fruit setting and the sucrose unloading capacity of young fruit. Plant Cell.

[56] Tang GQ, Sturm A (1999). Antisense repression of sucrose synthase in carrot (*Daucus carota* L.) affects growth rather than sucrose partitioning. Plant Mol Biol.

[57] Chourey PS (1981). Genetic control of sucrose synthetase in maize endosperm. Mol Gen Genet.

[58] Dale EMD, Housley TL (1986). Sucrose synthase activity in developing wheat endosperms differing in maximum weight. Plant Physiol.

[59] Kato T (1995). Changes of sucrose synthase activity in developing endosperm of rice cultivars. Crop Sci.

[60] Zhang S, Xie M, Ren G, Yu B (2013). CDC5, a DNA binding protein, positively regulates posttranscriptional processing and/or transcription of primary microRNA transcripts. Proc Natl Acad Sci U S A.

[61] Roudier F, Fernandez AG, Fujita M, Himmelspach R, Borner GHH, Schindelman G (2005). COBRA, an Arabidopsis extracellular glycosyl-phosphatidyl inositol-anchored protein, specifically controls highly anisotropic expansion through its involvement in cellulose microfibril orientation. Plant Cell.

[62] Sorek N, Sorek H, Kijac A, Szemenyei HJ, Bauer S, Hématy K (2014). The Arabidopsis COBRA protein facilitates cellulose crystallization at the plasma membrane. J Biol Chem.

[63] Dai XX, You CJ, Chen GX, Li XH, Zhang QF, Wu CY (2011). OsBC1L4 encodes a COBRA-like protein that affects cellulose synthesis in rice. Plant Mol Biol.

[64] Hochholdinger F, Wen TJ, Zimmermann R, Marolle PC, Silva ODC, Bruce W (2008). The maize (*Zea mays* L.) roothairless3 gene encodes a putative GPI-anchored, monocot-specific, COBRA-like protein that significantly affects grain yield. Plant J.

