# Supplementary material for: Genome-wide sRNA and mRNA transcriptomic profiling insights into dynamic regulation of taproot thickening in radish (Raphanus sativus L.)
Source: BMC Plant Biol. 2020 Aug 8;20:373. doi: 10.1186/s12870-020-02585-z (PMC7414755; doi:10.1186/s12870-020-02585-z)
Supplement: Supplementary file 1 — Additional file 1 Figure S1. The sequence length distribution of small RNAs during radish taproot thickening. Figure S2. The heatmap of DEGs and DEMs in radish taproot thickening. a. Heatmap of DEMs in ‘NAU-DY’; b, c represents heatmap of DEGs in ‘NAU-DY’ and ‘NAU-YB’, respectively. Figure S3. Comparative analysis of gene expression profiles from RNA-seq and RT-qPCR. 20 randomly selected genes by RNA-seq and RT-qPCR showing different expression patterns in three comparative groups (DS2 vs DS1, DS3 vs DS1, and DS3 vs DS2) during taproot thickening. Each data point represents the log2 normalized expression level obtained from RNA-seq (x axis) and RT-qPCR (y axis) analyses. Figure S4. RT-qPCR validation of 14 DEMs during radish taproot thickening. The relative expression of DEMs between DS2 and DS1 libraries (a), DS3 and DS1 libraries (b) and DS3 and DS2 libraries (c) were analyzed by the 2−ΔΔCT method. Table S1. Primer sequences for RT-qPCR assay. Table S2. Summary of small RNA sequencing data. Table S3. Summary of mRNA sequencing data. Table S4. Detailed information of DEMs during radish taproot thickening in ‘NAU-DY’. Table S6. DEMs and their corresponding targets extracted from RNA-seq. [file 12870_2020_2585_MOESM1_ESM.docx]

**Genome-wide sRNA and mRNA** **Transcriptomic Profiling Insights into** **Dynamic Regulation of Taproot Thickening in Radish (*Raphanus sativus* L.)**

Yang Xie^1,2^, Jiali Ying^1^, Liang Xu^1^, Yan Wang^1^, Junhui Dong^1^, Yinglong Chen^3^, Mingjia Tang^1^, Cui Li^1^, Everlyne M’mbone Muleke^1^, Liwang Liu^1,*^


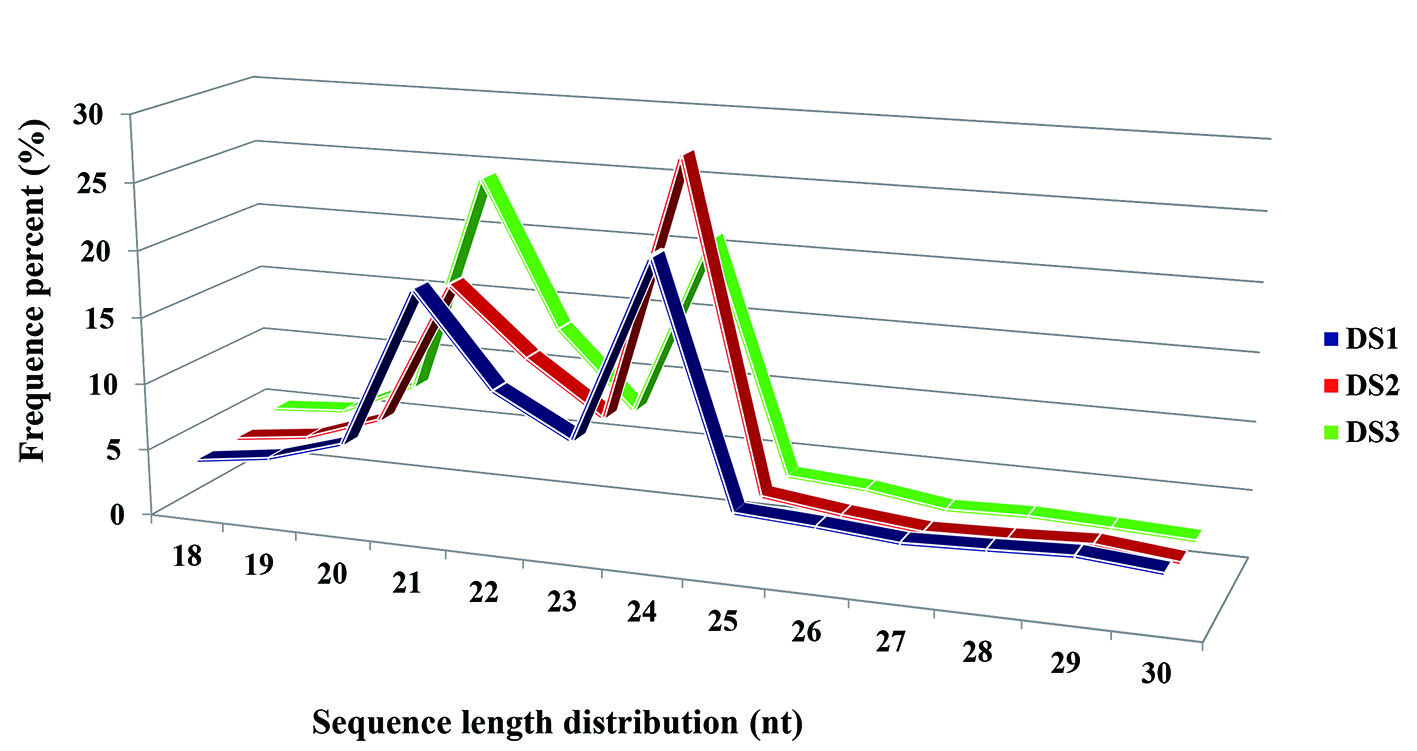


Figure S1 The sequence length distribution of small RNAs during radish taproot thickening.


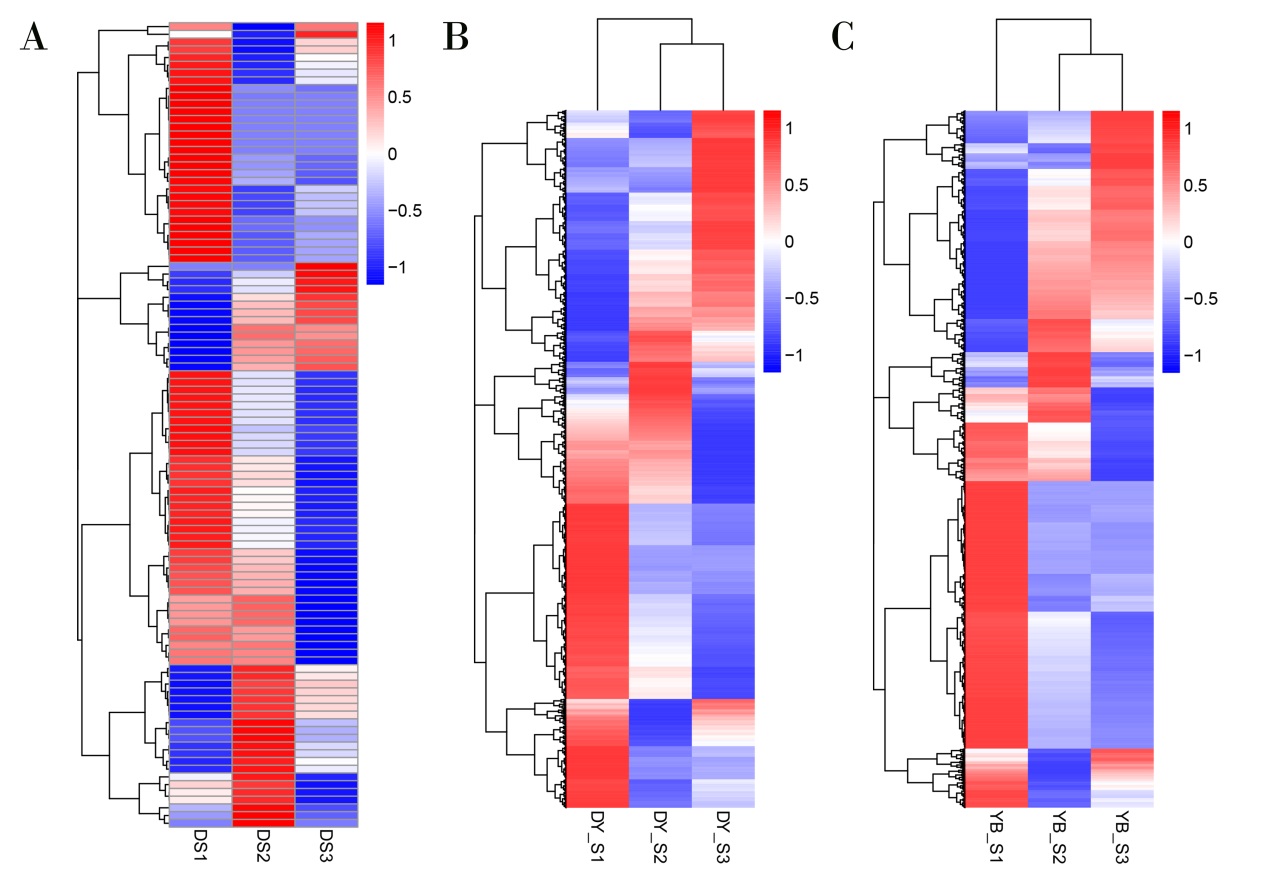


Figure S2 The heatmap of DEGs and DEMs in radish taproot thickening. a. Heatmap of DEMs in ‘NAU-DY’; b, c reprensents heatmap of DEGs in ‘NAU-DY’ and ‘NAU-YB’, respectively.

Figure S3 Comparative analysis of gene expression profiles from RNA-seq and RT-qPCR. 16 randomly selected genes by RNA-seq and RT-qPCR showing different expression patterns in three comparative groups (DS2 vs DS1, DS3 vs DS1, and DS3 vs DS2) during taproot thickening. Each data point represents the log_2_ normalized expression level obtained from RNA-seq (x axis) and RT-qPCR (y axis) analyses.


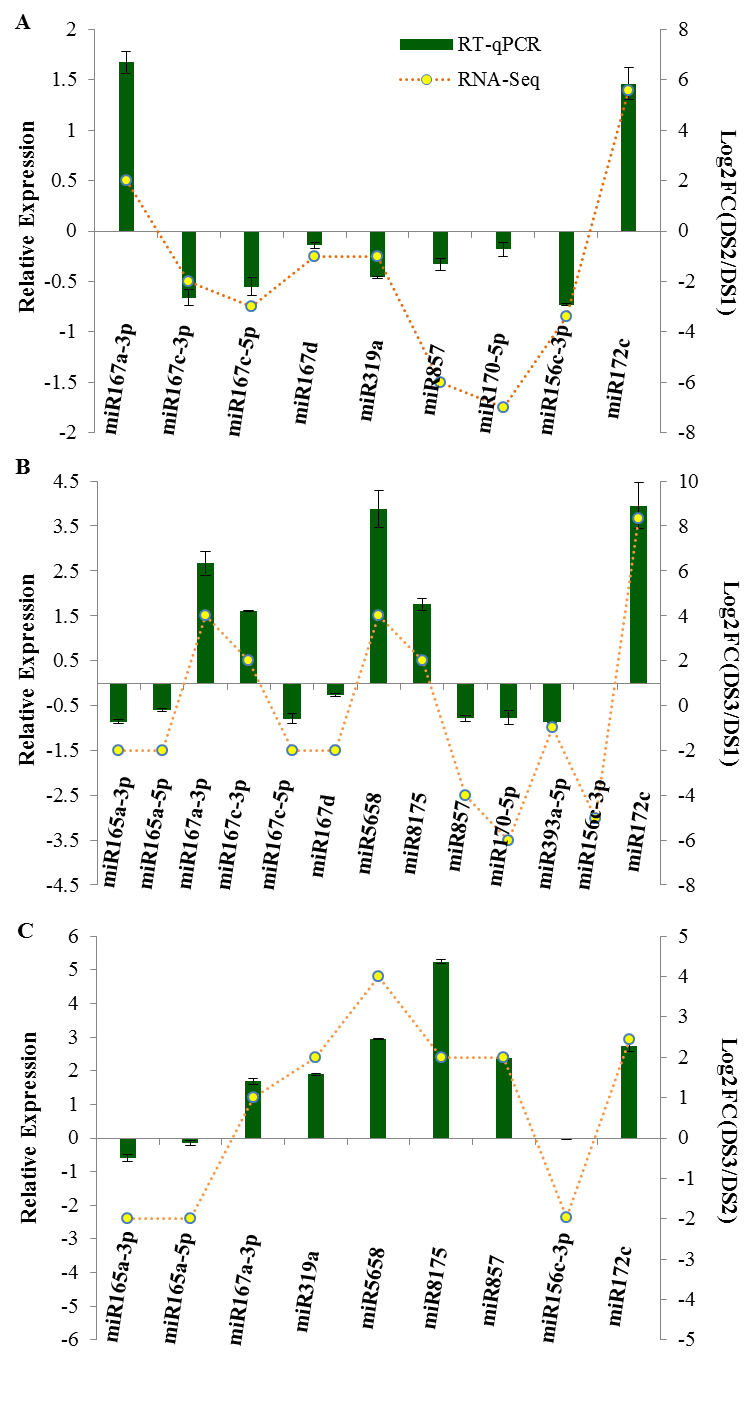


Figure S4. RT-qPCR validation of 14 DEMs during radish taproot thickening. The relative expression of DEMs between DS2 and DS1 libraries (a), DS3 and DS1 libraries (b) and DS3 and DS2 libraries (c) were analyzed by the 2^−ΔΔ^*^C^*^T^ method.

**Table S1 Primer sequences for RT-qPCR assay**

| **Primer name** | **Primer sequence** | **Gene ID** | **Notation** |
| --- | --- | --- | --- |
| *IAA26*-F | GAGAAGAGAAACCCATCAT | Rsa1.0_05596.1_g00001.1 | Auxin-responsive protein |
| *IAA26*-R | TTACTGCCAAGGAACATC |  |  |
| *AUX1*-F | GCTTCACCGTCTATATCAT | Rsa1.0_00328.1_g00005.1 | Auxin transporter protein 1 |
| *AUX1*-R | CCGAATCCGACTATTAGAA |  |  |
| *AIR12*-F | CTCCTCATTCTTCTCTTCTT | Rsa1.0_02513.1_g00004.1 | Auxin-induced in root cultures protein 12 |
| *AIR12*-R | CGTTGTAAGTGTAGTGGA |  |  |
| *PIN3*-F | AGAAACCCTAACACTTACTC | Rsa1.0_00699.1_g00018.1 | Auxin efflux carrier component 3 |
| *PIN3*-R | GCCAGCATCAGATAGAAT |  |  |
| *PPC2*-F | TACCGTTCTGTTGTCTTC | Rsa1.0_00647.1_g00001.1 | Phosphoenolpyruvate carboxylase 2 |
| *PPC2*-R | GTCTGCTTCCTATGTTCA |  |  |
| *CYP79F1*-F | CTCCTTGCTTACCTTCTC | Rsa1.0_00065.1_g00017.1 | Dihomomethionine N-hydroxylase |
| *CYP79F1*-R | ATCAATGTTCCAACCTCTAA |  |  |
| *GLT1*-F | ATTGGAGATGTTGATAAGGAT | Rsa1.0_00283.1_g00005.1 | Glutamate synthase 1 |
| *GLT1*-R | GAGCGTAGAAGTGATGAG |  |  |
| *AGPS1*-F | TGCTTGATGCTGATGTTA | Rsa1.0_00641.1_g00004.1 | Glucose-1-phosphate adenylyltransferase |
| *AGPS1*-R | ATTGTCCGTGTTGATGAT |  |  |
| *ASPG2*-F | GGAATAGCGATGATAATAACG | Rsa1.0_00082.1_g00007.1 | Aspartic protease in guard cell 2 |
| *ASPG2*-R | TGAACACAAGACGATGAA |  |  |
| *GSTF3*-F | GCGATTACTCAATACATAGC | Rsa1.0_04933.1_g00001.1 | Glutathione S-transferase F3 |
| *GSTF3*-R | CTAACTTAGCCTCCTCTTC |  |  |
| *APA1*-F | GATGAAGAAGGTGGTGAA | Rsa1.0_23081.1_g00001.1 | Aspartic proteinase A1 |
| *APA1*-R | CAGCAAGCAAAGATGTTC |  |  |
| *GLN1-2*-F | CATCGGTTGGTATCTCAG | Rsa1.0_02919.1_g00001.1 | Glutamine synthetase cytosolic isozyme 1-2 |
| *GLN1-2*-R | TATCTCGTATCCTCCTTCC |  |  |
| *AUR*-F | GCTCCTCATTCTTCTCTTC | Rsa1.0_00196.1_g00003.1 | Auxin-induced in root cultures protein |
| *AUR*-R | GTGGCGTTGTAAGTGTAG |  |  |
| *JAL34*-F | CGATGCTGTAACCAAGAT | Rsa1.0_00239.1_g00017.1 | Jacalin-related lectin 34 |
| *JAL34*-R | AGAAGAGTCATACCAACCT |  |  |
| *EXPB3*-F | ATTACAGTTGCCATCCTAC | Rsa1.0_00113.1_g00004.1 | Expansin B3 |
| *EXPB3*-R | GCCTCATATACCTATTCCTAC |  |  |
| *CDC5*-F | TCATCTTCGGTGTTCTTC | Rsa1.0_00867.1_g00008.1 | Cell division cycle 5-like protein |
| *CDC5*-R | CCTCTGTTCTTCGTTCTG |  |  |
| *SPS1*-F | GACTTACCTATCTCGCATAA | Rsa1.0_00123.1_g00072.1 | Sucrose-phosphate synthase 1 |
| *SPS1*-R | GGAGTTACCGTCTTGATT |  |  |
| *SUS1*-F | ATTGGAGATGGTGTTGAG | Rsa1.0_00483.1_g00003.1 | Sucrose synthase 1 |
| *SUS1*-R | GCTGTGTAGTTGAAGGAA |  |  |
| *BAM5*-F | GCACTCTCAACTTCACTT | Rsa1.0_00777.1_g00012.1 | Beta-amylase 5 |
| *BAM5*-R | TCACCAGCAACTTCTATG |  |  |
| *BFRUCT4* -F | TTGTTCATCGCCTTCTAC | Rsa1.0_00018.1_g00046.1 | Acid beta-fructofuranosidase 4 |
| *BFRUCT4* -R | ATCGTTCATCCAGTTCTG |  |  |
| *ARF8-F* | TCCGACAAGAAGTTAGTAA | Rsa1.0_00548.1_g00007.1 | Auxin response factor 8 |
| *ARF8-R* | GTTGTTGAAGTTGACCAT |  |  |
| *bHLH77-F* | GAGTCATTAACGCAGTCT | Rsa1.0_02710.1_g00004.1 | Transcription factor bHLH77 |
| *bHLH77-R* | TTGGCAAGAGAATAGTATCC |  |  |
| *APL-F* | ACGATAATGAGAGTGATG | Rsa1.0_00925.1_g00004.1 | Myb family transcription factor APL |
| *APL-R* | GGAGTAGTGAATACAACA |  |  |
| miR165a-3p | TCGGACCAGGCTTCATCCCCC | | |
| miR165a-5p | GGAATGTTGTCTGGATCGAGG | | |
| miR167a-3p | GATCATGTTCGCAGTTTCACC | | |
| miR167c-3p | TAGGTCATGCTGGTAGTTTCACC | | |
| miR167c-5p | TAAGCTGCCAGCATGATCTTG | | |
| miR167d | TGAAGCTGCCAGCATGATCTGG | | |
| miR319a | TTGGACTGAAGGGAGCTCCCT | | |
| miR5658 | ATGATGATGATGATGATGAAA | | |
| miR8175 | GATCCCCGGCAACGGCGCCA | | |
| miR857 | TTTTGTATGTTGAAGGTGTAT | | |
| miR170-5p | TATTGGCCTGGTTCACTCAGA | | |
| miR393a-5p  miR172c  miR156c-3p | TCCAAAGGGATCGCATTGATCC  AGAATCTTGATGATGCTGCAG  GCTCACTGCTCTATCTGTCAGA | | |

**Table S2 Summary of small RNA sequencing data results**

| **Sample** | **DS1** | **DS2** | **DS3** |
| --- | --- | --- | --- |
| Primary data |  |  |  |
| Raw reads | 15,239,556 (100%) | 16,161,768 (100%) | 17,569,639 (100%) |
| Clean reads (redundant) | 14,811,420 (97.19%) | 15,646,201 (96.81%) | 16,952,159 (96.48%) |
| Clean reads (unique) | 10,859,916 (100%) | 12,203,275 (100%) | 13,064,167 (100%) |
| Mapped genome (unique) | 7,557,902 (69.59%) | 9,581,912 (78.52%) | 10,554,810 (80.79%) |
| Known miRNAs | | | |
| Mapped uniq sRNA | 906 | 694 | 723 |
| Mapped total sRNA | 486,571 | 320,881 | 709,526 |
| Mapped known miRNAs | 80 | 71 | 74 |
| Novel miRNAs | | | |
| Mapped uniq sRNA | 775 | 748 | 711 |
| Mapped total sRNA | 39,049 | 59,626 | 97,877 |
| Mapped novel miRNAs | 42 | 41 | 38 |

**Table S3 Summary of mRNA sequencing data results**

| **Sample** | **YB_S1** | **YB_S2** | **YB_S3** | **DY_S1** | **DY_S2** | **DY_S3** |
| --- | --- | --- | --- | --- | --- | --- |
| Raw reads | 33,723,124 | 34,154,824 | 32,478,916 | 36,654,108 | 26,786,020 | 29,728,960 |
| Clean reads | 32,540,796 | 33,096,322 | 31,624,402 | 34,843,676 | 25,418,388 | 28,280,588 |
| Total mapped | 21,289,162 | 22,514,827 | 21,352,317 | 22,781,453 | 16,298,950 | 18,221,788 |
| Multiple mapped | 153,482 | 155,974 | 139,502 | 163,705 | 108,945 | 120,277 |
| Uniquely mapped | 21,135,680 | 22,358,853 | 21,212,815 | 22,617,748 | 16,190,005 | 18,101,511 |
| Read-1 | 11,550,114 | 12,048,362 | 11,525,923 | 12,547,871 | 9,234,008 | 10,223,896 |
| Read-2 | 9,585,566 | 10,310,491 | 9,686,892 | 10,069,877 | 6,955,997 | 7,877,615 |
| Reads map to '+' | 10,559,019 | 11,160,672 | 10,564,040 | 11,305,411 | 8,082,756 | 9,013,471 |
| Reads map to '-' | 10,576,661 | 11,198,181 | 10,648,775 | 11,312,337 | 8,107,249 | 9,088,040 |
| Non-splice reads | 13,504,979 | 14,296,018 | 13,241,374 | 14,449,717 | 10,299,910 | 11,292,084 |
| Splice reads | 7,630,701 | 8,062,835 | 7,971,441 | 8,168,031 | 5,890,095 | 6,809,427 |

**Table S4 Detailed information of DEMs during radish taproot thickening in ‘NAU-DY’**

| **sRNA** | **Standardized read count value** | | **log_2_FC (DS2/DS1)** | ***p*.value** | **Standardized read count value** | | **log_2_FC (DS3/DS1)** | ***p*.value** | **Standardized read count value** | | **log_2_FC (DS3/DS2)** | ***p*.value** |
| --- | --- | --- | --- | --- | --- | --- | --- | --- | --- | --- | --- | --- |
|  | **DS2** | **DS1** |  |  | **DS3** | **DS1** |  |  | **DS3** | **DS2** |  |  |
| **Cluster 1** |  |  |  |  |  |  |  |  |  |  |  |  |
| miR172c | 1943.09 | 40.60 | 5.58 | 0 | 9826.59 | 30.35 | 8.34 | 0 | 8598.88 | 1578.21 | 2.45 | 0 |
| novel_36 | 359.25 | 0.00 | 9.49 | 4.65E-64 | 3851.54 | 0.00 | 12.91 | 0 | 3370.34 | 291.79 | 3.53 | 0 |
| novel_5 | 23259.43 | 1396.97 | 4.06 | 0 | 66504.76 | 1044.35 | 5.99 | 0 | 58195.84 | 18891.64 | 1.62 | 0 |
| miR408-3p | 1342.60 | 8929.87 | -2.73 | 0 | 583.21 | 6675.84 | -3.52 | 0 | 510.35 | 1090.48 | -1.10 | 2.04E-106 |
| miR156a-5p | 584.76 | 4727.91 | -3.02 | 0 | 131.26 | 3534.51 | -4.75 | 0 | 114.87 | 474.95 | -2.05 | 2.80E-84 |
| miR172a | 215.03 | 70.13 | 1.62 | 5.05E-17 | 1068.40 | 52.42 | 4.35 | 1.88E-155 | 934.91 | 174.65 | 2.42 | 6.06E-79 |
| miR408-5p | 925.66 | 3613.29 | -1.96 | 0 | 427.03 | 2701.24 | -2.66 | 0 | 373.67 | 751.83 | -1.01 | 2.05E-68 |
| miR172e-3p | 1072.50 | 16.61 | 6.01 | 4.13E-248 | 2274.70 | 12.42 | 7.52 | 0 | 1990.51 | 871.10 | 1.19 | 7.87E-33 |
| miR157a-3p | 112.76 | 527.78 | -2.23 | 2.09E-69 | 3.32 | 394.56 | -6.89 | 4.92E-123 | 2.91 | 91.58 | -4.98 | 4.31E-28 |
| miR156c-3p | 159.96 | 1677.47 | -3.39 | 0 | 38.22 | 1254.05 | -5.04 | 0 | 33.44 | 129.92 | -1.96 | 2.03E-23 |
| miR396b-3p | 270.09 | 86.73 | 1.64 | 2.16E-21 | 702.85 | 64.84 | 3.44 | 9.62E-77 | 615.04 | 219.37 | 1.49 | 9.43E-20 |
| miR156a-3p | 73.42 | 3013.53 | -5.36 | 0 | 3.32 | 2252.87 | -9.41 | 0 | 2.91 | 59.64 | -4.36 | 1.93E-18 |
| novel_136 | 138.98 | 0.00 | 8.12 | 4.82E-30 | 39.88 | 0.00 | 6.32 | 9.60E-09 | 34.90 | 112.88 | -1.69 | 3.45E-18 |
| novel_80 | 65.56 | 177.16 | -1.43 | 1.72E-14 | 3.32 | 132.44 | -5.32 | 1.38E-46 | 2.91 | 53.25 | -4.19 | 1.84E-16 |
| miR858a | 68.18 | 710.48 | -3.38 | 2.72E-139 | 259.21 | 531.14 | -1.04 | 3.18E-79 | 226.82 | 55.38 | 2.03 | 3.14E-15 |
| novel_29 | 97.02 | 11.07 | 3.13 | 9.20E-18 | 39.88 | 8.28 | 2.27 | 0.0036907 | 34.90 | 78.80 | -1.18 | 1.11E-09 |
| novel_122 | 31.47 | 12.92 | 1.28 | 0.0076574 | 3.32 | 9.66 | -1.54 | 0.0033804 | 2.91 | 25.56 | -3.14 | 1.07E-07 |
| novel_73 | 15.73 | 256.51 | -4.03 | 9.27E-58 | 0.00 | 191.76 | -8.58 | 2.24E-50 | 0.00 | 12.78 | -4.68 | 4.35E-05 |
| miR171a-3p | 28.84 | 398.61 | -3.79 | 1.47E-85 | 11.63 | 297.99 | -4.68 | 5.08E-103 | 10.18 | 23.43 | -1.20 | 0.0007799 |
| miR171b-3p | 23.60 | 145.79 | -2.63 | 3.80E-24 | 9.97 | 108.99 | -3.45 | 2.63E-36 | 8.72 | 19.17 | -1.14 | 0.003123 |
| miR169b-3p | 20.98 | 101.50 | -2.27 | 5.42E-15 | 8.31 | 75.88 | -3.19 | 5.23E-25 | 7.27 | 17.04 | -1.23 | 0.0037884 |
| miR171b-5p | 7.87 | 38.75 | -2.30 | 1.16E-06 | 0.00 | 28.97 | -5.86 | 3.28E-11 | 0.00 | 6.39 | -3.68 | 0.0054863 |
| novel_100 | 7.87 | 90.42 | -3.52 | 8.76E-20 | 0.00 | 67.60 | -7.08 | 3.70E-22 | 0.00 | 6.39 | -3.68 | 0.0054863 |
| novel_121 | 13.11 | 35.06 | -1.42 | 0.00070718 | 4.98 | 26.21 | -2.39 | 1.95E-08 | 4.36 | 10.65 | -1.29 | 0.019199 |
| novel_90 | 13.11 | 273.12 | -4.38 | 5.44E-64 | 33.23 | 204.18 | -2.62 | 2.50E-58 | 29.08 | 10.65 | 1.45 | 0.055924 |
| miR857 | 2.62 | 199.30 | -6.25 | 1.17E-49 | 8.31 | 149.00 | -4.16 | 1.07E-51 | 7.27 | 2.13 | 1.77 | 0.22263 |
| miR858b | 2.62 | 33.22 | -3.66 | 2.21E-08 | 8.31 | 24.83 | -1.58 | 2.09E-06 | 7.27 | 2.13 | 1.77 | 0.22263 |
| miR397a | 10.49 | 557.31 | -5.73 | 1.20E-136 | 19.94 | 416.64 | -4.39 | 2.16E-142 | 17.45 | 8.52 | 1.03 | 0.38161 |
| **Cluster 2** |  |  |  |  |  |  |  |  |  |  |  |  |
| miR159a | 234589.80 | 103207.50 | 1.18 | 0 | 235274.81 | 77156.40 | 1.61 | 0 | N | N | N | N |
| miR159b-3p | 43791.70 | 13445.56 | 1.70 | 0 | 38232.97 | 10051.70 | 1.93 | 0 | N | N | N | N |
| miR160a-5p | 1067.26 | 2790.24 | -1.39 | 2.04E-194 | 805.87 | 2085.94 | -1.37 | 0 | N | N | N | N |
| miR394a | 13929.43 | 4331.15 | 1.69 | 0 | 12171.08 | 3237.90 | 1.91 | 0 | N | N | N | N |
| miR396b-5p | 81630.88 | 27983.60 | 1.54 | 0 | 76642.06 | 20920.12 | 1.87 | 0 | N | N | N | N |
| miR398b-3p | 2207.94 | 11221.86 | -2.35 | 0 | 3927.98 | 8389.29 | -1.09 | 0 | N | N | N | N |
| novel_25 | 3970.10 | 8570.02 | -1.11 | 0 | 2165.04 | 6406.82 | -1.57 | 0 | N | N | N | N |
| novel_38 | 78864.40 | 17143.74 | 2.20 | 0 | 56148.13 | 12816.40 | 2.13 | 0 | N | N | N | N |
| novel_1 | 713.25 | 2607.55 | -1.87 | 1.98E-271 | 882.30 | 1949.36 | -1.14 | 1.51E-306 | N | N | N | N |
| miR157a-5p | 26.22 | 1210.58 | -5.53 | 3.59E-294 | 14.95 | 905.01 | -5.92 | 6.30E-300 | N | N | N | N |
| novel_37 | 359.25 | 1326.84 | -1.88 | 2.03E-140 | 191.08 | 991.93 | -2.38 | 2.01E-260 | N | N | N | N |
| miR159c | 11556.29 | 4237.03 | 1.45 | 0 | 9984.44 | 3167.54 | 1.66 | 8.14E-174 | N | N | N | N |
| miR391-5p | 647.70 | 105.19 | 2.62 | 1.11E-90 | 1104.95 | 78.64 | 3.81 | 3.95E-138 | N | N | N | N |
| miR395a | 610.99 | 59.05 | 3.37 | 5.19E-110 | 697.86 | 44.15 | 3.98 | 8.21E-93 | N | N | N | N |
| miR167c-5p | 41.96 | 332.17 | -2.99 | 1.20E-59 | 58.16 | 248.33 | -2.09 | 1.42E-61 | N | N | N | N |
| novel_95 | 0.00 | 190.08 | -8.57 | 5.94E-40 | 0.00 | 142.10 | -8.15 | 6.94E-40 | N | N | N | N |
| novel_111 | 0.00 | 167.93 | -8.39 | 3.47E-36 | 0.00 | 125.54 | -7.97 | 3.42E-36 | N | N | N | N |
| miR167c-3p | 39.33 | 182.69 | -2.22 | 5.19E-25 | 41.54 | 136.58 | -1.72 | 2.00E-30 | N | N | N | N |
| novel_119 | 0.00 | 77.51 | -7.28 | 2.50E-19 | 3.32 | 57.94 | -4.12 | 4.34E-21 | N | N | N | N |
| novel_120 | 0.00 | 75.66 | -7.24 | 5.98E-19 | 0.00 | 56.56 | -6.82 | 3.27E-19 | N | N | N | N |
| miR170-5p | 0.00 | 57.21 | -6.84 | 4.69E-15 | 0.00 | 42.77 | -6.42 | 2.40E-15 | N | N | N | N |
| miR169g-3p | 20.98 | 71.97 | -1.78 | 1.54E-08 | 11.63 | 53.80 | -2.21 | 4.36E-15 | N | N | N | N |
| miR169b-5p | 5.24 | 57.21 | -3.45 | 6.87E-13 | 4.98 | 42.77 | -3.10 | 1.33E-14 | N | N | N | N |
| miR167a-3p | 62.93 | 12.92 | 2.28 | 7.08E-09 | 112.99 | 9.66 | 3.55 | 2.72E-14 | N | N | N | N |
| miR157c-3p | 0.00 | 44.29 | -6.47 | 3.49E-12 | 0.00 | 33.11 | -6.05 | 1.74E-12 | N | N | N | N |
| miR828 | 0.00 | 36.91 | -6.21 | 1.78E-10 | 0.00 | 27.59 | -5.79 | 8.88E-11 | N | N | N | N |
| miR157d | 0.00 | 23.99 | -5.58 | 2.46E-07 | 0.00 | 17.93 | -5.16 | 1.27E-07 | N | N | N | N |
| novel_96 | 83.91 | 23.99 | 1.81 | 1.25E-08 | 101.36 | 17.93 | 2.50 | 1.96E-07 | N | N | N | N |
| miR169a-5p | 2.62 | 20.30 | -2.95 | 6.17E-05 | 0.00 | 15.18 | -4.92 | 1.14E-06 | N | N | N | N |
| miR827 | 0.00 | 16.61 | -5.05 | 1.99E-05 | 3.32 | 12.42 | -1.90 | 0.0003403 | N | N | N | N |
| miR395b | 243.87 | 44.29 | 2.46 | 7.76E-33 | 116.31 | 33.11 | 1.81 | 0.0003668 | N | N | N | N |
| miR172b-5p | 0.00 | 18.45 | -5.21 | 6.48E-06 | 4.98 | 13.80 | -1.47 | 0.0005728 | N | N | N | N |
| novel_51 | 1101.35 | 350.63 | 1.65 | 6.10E-83 | 623.09 | 262.12 | 1.25 | 0.0006667 | N | N | N | N |
| **Cluster 3** |  |  |  |  |  |  |  |  |  |  |  |  |
| miR158a-3p | 111186.35 | 54439.20 | 1.03 | 0 | N | N | N | N | 38257.31 | 90307.14 | -1.24 | 0 |
| miR319a | 162378.06 | 373419.71 | -1.20 | 0 | N | N | N | N | 475334.63 | 131885.78 | 1.85 | 0 |
| miR824-3p | 999.08 | 3092.88 | -1.63 | 6.30E-269 | N | N | N | N | 2010.86 | 811.47 | 1.31 | 1.89E-43 |
| novel_123 | 249.11 | 51.67 | 2.27 | 1.88E-30 | N | N | N | N | 39.26 | 202.33 | -2.37 | 1.61E-41 |
| novel_109 | 57.69 | 3.69 | 3.97 | 3.08E-13 | N | N | N | N | 10.18 | 46.86 | -2.20 | 2.88E-10 |
| novel_141 | 204.54 | 411.52 | -1.01 | 1.69E-19 | N | N | N | N | 396.94 | 166.13 | 1.26 | 7.59E-09 |
| novel_129 | 28.84 | 3.69 | 2.97 | 5.21E-06 | N | N | N | N | 4.36 | 23.43 | -2.43 | 3.50E-06 |
| novel_135 | 7.87 | 0.00 | 3.98 | 0.0070526 | N | N | N | N | 1.45 | 6.39 | -2.14 | 0.021583 |
| **Cluster 4** |  |  |  |  |  |  |  |  |  |  |  |  |
| miR165a-3p | N | N | N | N | 4218.75 | 12832.96 | -1.61 | 0 | 3691.67 | 11786.51 | -1.67 | 0 |
| novel_33 | N | N | N | N | 1129.87 | 2599.15 | -1.20 | 0 | 988.71 | 3115.95 | -1.66 | 0 |
| novel_44 | N | N | N | N | 372.19 | 1066.42 | -1.52 | 1.93E-206 | 325.69 | 1601.64 | -2.30 | 0 |
| novel_60 | N | N | N | N | 93.05 | 608.40 | -2.71 | 2.82E-173 | 81.42 | 615.52 | -2.92 | 3.20E-143 |
| miR164c-3p | N | N | N | N | 443.64 | 1397.53 | -1.66 | 4.58E-287 | 388.21 | 828.51 | -1.09 | 3.55E-81 |
| miR165a-5p | N | N | N | N | 83.08 | 295.23 | -1.83 | 1.42E-66 | 72.70 | 315.22 | -2.12 | 3.78E-58 |
| miR167a-5p | N | N | N | N | 1880.91 | 546.32 | 1.78 | 3.50E-44 | 1645.91 | 760.35 | 1.11 | 2.80E-22 |
| miR8175 | N | N | N | N | 407.09 | 140.72 | 1.53 | 1.43E-06 | 356.23 | 102.23 | 1.80 | 3.41E-18 |
| miR156b-3p | N | N | N | N | 26.59 | 121.40 | -2.19 | 7.01E-32 | 23.26 | 70.28 | -1.60 | 2.56E-11 |
| novel_116 | N | N | N | N | 0.00 | 28.97 | -5.86 | 3.28E-11 | 0.00 | 19.17 | -5.26 | 4.83E-07 |
| novel_124 | N | N | N | N | 3.32 | 28.97 | -3.12 | 2.14E-10 | 2.91 | 19.17 | -2.72 | 1.16E-05 |
| novel_133 | N | N | N | N | 3.32 | 12.42 | -1.90 | 0.0003403 | 2.91 | 19.17 | -2.72 | 1.16E-05 |
| miR160a-3p | N | N | N | N | 4.98 | 15.18 | -1.61 | 0.0001908 | 4.36 | 21.30 | -2.29 | 1.57E-05 |
| novel_85 | N | N | N | N | 343.95 | 97.95 | 1.81 | 9.13E-10 | 300.98 | 144.83 | 1.06 | 0.0001623 |
| miR156g | N | N | N | N | 0.00 | 19.31 | -5.27 | 4.35E-08 | 0.00 | 10.65 | -4.41 | 0.0002085 |
| miR5658 | N | N | N | N | 6.65 | 0.00 | 3.73 | 0.06 | 5.82 | 0.00 | 3.54 | 0.051848 |
| miR390b-3p | N | N | N | N | 6.65 | 0.00 | 3.73 | 0.06 | 5.82 | 2.13 | 1.45 | 0.39 |
| **Cluster 5** |  |  |  |  |  |  |  |  |  |  |  |  |
| novel_39 | 936.15 | 2530.04 | -1.43 | 5.15E-185 | N | N | N | N | N | N | N | N |
| miR167d | 676.54 | 1452.33 | -1.10 | 1.43E-73 | N | N | N | N | N | N | N | N |
| miR824-5p | 217.65 | 682.80 | -1.65 | 9.60E-62 | N | N | N | N | N | N | N | N |
| miR398a-3p | 102.27 | 245.44 | -1.26 | 1.67E-16 | N | N | N | N | N | N | N | N |
| miR162a-5p | 272.71 | 131.02 | 1.06 | 3.20E-11 | N | N | N | N | N | N | N | N |
| novel_144 | 57.69 | 18.45 | 1.64 | 1.08E-05 | N | N | N | N | N | N | N | N |
| miR160c-3p | 0.00 | 9.23 | -4.21 | 0.0021119 | N | N | N | N | N | N | N | N |
| miR400 | 13.11 | 31.37 | -1.26 | 0.0032858 | N | N | N | N | N | N | N | N |
| **Cluster 6** |  |  |  |  |  |  |  |  |  |  |  |  |
| miR162a-3p | N | N | N | N | 55466.88 | 26431.59 | 1.07 | 7.69E-56 | N | N | N | N |
| miR399b | N | N | N | N | 31.57 | 96.57 | -1.61 | 4.24E-21 | N | N | N | N |
| novel_142 | N | N | N | N | 6.65 | 16.56 | -1.32 | 0.0002875 | N | N | N | N |
| miR169f-3p | N | N | N | N | 4.98 | 13.80 | -1.47 | 0.0005728 | N | N | N | N |
| miR393a-5p | N | N | N | N | 4.98 | 13.80 | -1.47 | 0.0005728 | N | N | N | N |
| miR166e-5p | N | N | N | N | 0.00 | 6.90 | -3.79 | 0.0012982 | N | N | N | N |
| novel_46 | N | N | N | N | 3.32 | 6.90 | -1.05 | 0.030708 | N | N | N | N |
| **Cluster 7** |  |  |  |  |  |  |  |  |  |  |  |  |
| miR158b | N | N | N | N | N | N | N | N | 190.47 | 513.29 | -1.43 | 1.28E-65 |
| miR166a-5p | N | N | N | N | N | N | N | N | 155.58 | 411.06 | -1.40 | 6.34E-52 |
| miR390a-3p | N | N | N | N | N | N | N | N | 1.45 | 6.39 | -2.14 | 0.021583 |

**Table S6 DEMs and their corresponding targets extracted from RNA-seq**

| **miRNA** | **Target** | **Gene name** | **Annotation** |
| --- | --- | --- | --- |
| **S1 vs S2** | | | |
| miR156c-3p | Rsa1.0_00868.1_g00005.1 | - | Alpha/beta hydrolase |
| miR159b-3p | Rsa1.0_05577.1_g00002.1 | ZCF37 |  |
| miR170-5p | Rsa1.0_05832.1_g00002.1 | BGAL4 | Glycoside hydrolase |
| miR172e-3p | Rsa1.0_01063.1_g00001.1 | RAP2.7 | DNA-binding, integrase-type |
| miR5658 | Rsa1.0_00451.1_g00004.1 | REN1 | Ternary complex factor MIP1 |
| novel_136 | Rsa1.0_00865.1_g00012.1 | PDR5 | Plant PDR ABC transporter associated |
| novel_136 | Rsa1.0_03188.1_g00002.1 | AAE7 | AMP-dependent synthetase/ligase |
| **S1 vs S3** | | | |
| miR157a-5p | Rsa1.0_01004.1_g00012.1 | - | Protein kinase |
| miR157d | Rsa1.0_04786.1_g00004.1 | SPL10 | Transcription factor, SBP-box |
| miR158b | Rsa1.0_03991.1_g00002.1 | FUT2 | Xyloglucan fucosyltransferase |
| miR167c-5p | Rsa1.0_00548.1_g00007.1 | ARF8 | DNA-binding pseudobarrel domain |
| miR169b-5p | Rsa1.0_00307.1_g00009.1 | NF-YA6 | CCAAT-binding factor |
| miR393a-5p | Rsa1.0_02710.1_g00004.1 | BHLH77 | Myc-type, basic helix-loop-helix (bHLH) domain |
| miR395b | Rsa1.0_00159.1_g00011.1 | - | - |
| miR395b | Rsa1.0_00381.1_g00016.1 | FER | Malectin-like carbohydrate-binding domain |
| miR397a | Rsa1.0_00328.1_g00010.1 | IRX12 | Laccase |
| miR397a | Rsa1.0_00333.1_g00009.1 | - | Protein kinase-like domain |
| miR397a | Rsa1.0_03355.1_g00004.1 | IRX12 | Laccase |
| miR408-5p | Rsa1.0_00341.1_g00034.1 | GH3.5 | GH3 auxin-responsive promoter |
| miR5658 | Rsa1.0_00657.1_g00003.1 | - | ATP-citrate lyase |
| miR5658 | Rsa1.0_00925.1_g00004.1 | APL | MYB-CC type transcription factor |
| miR5658 | Rsa1.0_03334.1_g00005.1 | GPX6 | Thioredoxin-like fold |
| miR824-3p | Rsa1.0_00001.1_g00031.1 | - | Carbohydrate-binding-like fold |
| miR828 | Rsa1.0_00685.1_g00004.1 | IQD5 | IQ motif, EF-hand binding site |
| novel_119 | Rsa1.0_01474.1_g00001.1 | NIP6;1 | Aquaporin-like\|Aquaporin-like |
| novel_129 | Rsa1.0_05003.1_g00001.1 | SYTE | C2 calcium-dependent membrane targeting |
| novel_136 | Rsa1.0_04683.1_g00003.1 | SPL11 | Transcription factor, SBP-box |
| novel_25 | Rsa1.0_00204.1_g00017.1 | BGLU40 | Glycoside hydrolase |
| novel_25 | Rsa1.0_01895.1_g00005.1 | PAP1 | Phosphatidic acid phosphatase type 2 |
| novel_29 | Rsa1.0_00022.1_g00024.1 | - | - |
| novel_36 | Rsa1.0_00144.1_g00004.1 | KIPK | Protein kinase-like domain |
| novel_38 | Rsa1.0_00404.1_g00018.1 | GATA11 | Zinc finger, GATA-type |
| novel_85 | Rsa1.0_00911.1_g00005.1 | RDR1 | RNA-dependent RNA polymerase |
| novel_85 | Rsa1.0_01651.1_g00001.1 | BHLH129 | Myc-type, basic helix-loop-helix (bHLH) domain |
| **S2 vs S3** | | | |
| miR398b-3p | Rsa1.0_09097.1_g00002.1 | RPL31C | Ribosomal protein L31e domain |
| miR857 | Rsa1.0_00451.1_g00023.1 | ATGSTU25 | Thioredoxin-like fold |
| novel_25 | Rsa1.0_00272.1_g00008.1 | CYCA1;1 | Cyclin A/B/D/E |
| novel_46 | Rsa1.0_00047.1_g00008.1 | - | Protein of unknown function DUF1218 |
| **S2 vs S1&S3 vs S1** | | | |
| miR157a-5p | Rsa1.0_00124.1_g00033.1 | - | Peptidase S8 |
| miR157a-5p | Rsa1.0_00894.1_g00013.1 | BCA4 | Carbonic anhydrase |
| miR165a-5p | Rsa1.0_00502.1_g00017.1 | HIPL1 | Glucose/Sorbosone dehydrogenase |
| miR165a-5p | Rsa1.0_00662.1_g00018.1 | - | FAD-binding, type 2 |
| miR167c-5p | Rsa1.0_00433.1_g00004.1 | PATL2 | CRAL/TRIO, N-terminal domain |
| miR169b-5p | Rsa1.0_00017.1_g00025.1 | WRKY11 | DNA-binding WRKY |
| miR169f-3p | Rsa1.0_02703.1_g00003.1 | PER45 | Peroxidase |
| miR172e-3p | Rsa1.0_00099.1_g00005.1 | TOE2 | AP2/ERF domain |
| miR172e-3p | Rsa1.0_04622.1_g00002.1 | ATTPS8 | HAD-like domain |
| miR395a | Rsa1.0_00468.1_g00009.1 | APS3 | ATP-sulfurylase PUA-like domain |
| miR395a | Rsa1.0_01524.1_g00007.1 | APS1 | ATP-sulfurylase |
| miR395b | Rsa1.0_00493.1_g00009.1 | AST68 | Sulphate anion transporter |
| miR828 | Rsa1.0_02069.1_g00002.1 | K2 | Kinesin, motor region |
| miR858a | Rsa1.0_01367.1_g00003.1 | MYB51 | Myb domain |
| novel_109 | Rsa1.0_05412.1_g00002.1 | - | Nucleotide-diphospho-sugar transferases |
| novel_111 | Rsa1.0_01373.1_g00003.1 | HAM1 | Transcription factor GRAS |
| novel_129 | Rsa1.0_00586.1_g00006.1 | MLP28 | Major latex protein domain |
| novel_141 | Rsa1.0_02387.1_g00004.1 | GSTU13 | Thioredoxin-like fold |
| novel_25 | Rsa1.0_00008.1_g00015.1 | LBD41 | Lateral organ boundaries, LOB |
| novel_25 | Rsa1.0_00077.1_g00004.1 | ARA1 | Ribosomal protein S5 domain 2-type fold |
| novel_37 | Rsa1.0_01835.1_g00003.1 | RHL41 | C2H2\|Zinc finger, C2H2 |
| **S2 vs S1&S3 vs S2** | | | |
| miR156c-3p | Rsa1.0_00093.1_g00005.1 | - | Pectin lyase fold |
| miR408-5p | Rsa1.0_03833.1_g00002.1 | ASP2 | Pyridoxal phosphate-dependent transferase |
| **S3 vs S1&S3 vs S2** | | | |
| miR165a-5p | Rsa1.0_00047.1_g00018.1 | CSLD2 | Cellulose synthase |
| miR167a-3p | Rsa1.0_03114.1_g00003.1 | BXL7 | Fibronectin type III-like domain |
| miR169b-5p | Rsa1.0_01855.1_g00009.1 | HAP2C | CCAAT-binding factor |
| miR169g-3p | Rsa1.0_01092.1_g00004.1 | - | Leucine-rich repeat-containing N-terminal |
| miR5658 | Rsa1.0_09640.1_g00001.1 | - | Zinc finger |
| novel_39 | Rsa1.0_03312.1_g00001.1 | XIG | Dil domain\|Myosin |
| novel_46 | Rsa1.0_04886.1_g00003.1 | BRL3 | Protein kinase |
| novel_73 | Rsa1.0_03073.1_g00005.1 | ARF17 | DNA-binding pseudobarrel domain |
| novel_85 | Rsa1.0_00566.1_g00003.1 | APX1 | Peroxidase |
| novel_85 | Rsa1.0_01921.1_g00003.1 | TGG2 | Glycoside hydrolase |
